# Stress-NRF2 response axis polarizes tumor macrophages and undermines immunotherapy

**DOI:** 10.1136/jitc-2025-013063

**Published:** 2025-10-31

**Authors:** Dominik J Schaer, Nadja Schulthess-Lutz, Livio Baselgia, Kahrisan Kunasingam, Rok Humar, Kerstin Hansen, Melanie Eschment, Elena Duerst, Florence Vallelian

**Affiliations:** 1 Department of Internal Medicine, University of Zurich, Zürich, Switzerland

**Keywords:** Macrophage, Tumor microenvironment - TME, Immunotherapy

## Abstract

**Background:**

Tumor-associated macrophages (TAMs) can switch between immune-activating and cancer-promoting states; yet, the stress pathways that lock them into procancerous states remain obscure. Here we defined the role of transcription factor NRF2 as a mediator of procancerous macrophages.

**Methods:**

We combined spatial transcriptomics, single-cell RNA sequencing, three-dimensional (3D) cell culture and in vivo tumor models to explore how NRF2 activation status in tumor-associated macrophages modifies responses to immunotherapy.

**Results:**

In MC38 colon tumors, repeated anti-CD40 or radiotherapy created necrosis that split TAMs into peripheral Cxcl9+ and peri-necrotic Spp1+ subsets. Spatial transcriptomics, single-cell RNA sequencing, and Keap1-deficient mice showed that the latter are NRF2-imprinted “stress-TAMs”, with immunosuppressive and tumor-promoting activity. The same NRF2 activation gradient separates pro-inflammatory CXCL9+ and anti-inflammatory SPP1+TAMs across diverse human cancers. NRF2-imprinted TAMs silence IFN-STAT1 programs, lose major histocompatibility complex-II and chemokine expression, fail to expand T cells, drive tumor cell invasion in 3D co-cultures, and foster metastasis. Constitutive hematopoietic NRF2 activation accelerated the growth of therapy-naïve MMTV-PyMT breast tumors and markedly impaired the efficacy of agonistic anti-CD40 antibody therapy in MC38 subcutaneous and lung-metastasis models. Conversely, macrophage-specific Nrf2 deletion restored immunogenic TAMs and potentiated anti-CD40 and anti-programmed cell death protein-1 treatments.

**Conclusions:**

Our data pinpoint a previously underappreciated cytoprotective mechanism, which inadvertently sustains immunosuppressive macrophages and confers therapy resistance. These results define stress-induced TAMs as an untapped driver of macrophage-based immune evasion. Inhibiting NRF2 activity alongside standard immunotherapies could restore a pro-inflammatory macrophage–T-cell amplification loop, potentially improving patient responses to T-cell—and macrophage-directed immunotherapies.

**WHAT IS ALREADY KNOWN ON THIS TOPIC**

- Tumor-associated macrophages (TAMs) exist along a spectrum from pro-inflammatory, antigen-presenting to immunosuppressive, influencing tumor behavior.
- Stressors common in the tumor microenvironment—oxidative stress, hypoxia, acidosis and hemorrhage—push TAMs toward immunosuppressive states that hinder T-cell infiltration and function.
- Transcription factor NRF2 confers resistance to oxidative damage but simultaneously dampens pro-inflammatory and antitumor activities of macrophages.

**WHAT THE STUDY ADDS**

- Immunotherapy-induced necrosis creates a spatial gradient from pro-inflammatory macrophages at the tumor margin towards NRF2-imprinted immunosuppressive TAMs in the necrotic core.
- NRF2 locks macrophages into a phenotype with impaired antigen presentation and T-cell recruitment capacity, accelerating tumor cell invasion and metastasis.
- NRF2 deletion in macrophages improves the efficacy of anti-CD40 and anti-programmed cell death protein-1 immunotherapies.

**HOW THIS STUDY MIGHT AFFECT RESEARCH, PRACTICE AND POLICY**

- This research provides a mechanistic framework of how NRF2 enhances macrophage heterogeneity and therapy resistance across cancer models.
- Development and implementation of macrophage-targeted NRF2 inhibitors may enhance antitumor immunity and extend response durability.

## Background

 Tumors are heterogeneous ecosystems where diverse immune cell populations coexist and interact within distinct “niches”. Among these, tumor-associated macrophages (TAMs) are often abundant in solid tumors, showing diversified phenotypes, transcriptional programs, and functions.^1^ TAMs can either be pro-inflammatory, promoting immune-mediated tumor regression, or adopt immunosuppressive programs that foster cancer progression. These opposing polarization states clinically correlate with disease outcomes in multiple malignancies.^2^ Despite ongoing efforts to therapeutically reprogram endogenous TAMs or stem-cell-derived engineered macrophages into cancer-fighting effectors, the specific signaling mechanisms that subvert pro-inflammatory macrophage states remain poorly characterized.^3 4^ This challenge prompts a central question: How do therapy-induced changes in the tumor microenvironment (TME) shape distinct macrophage subsets, and which pathways can we manipulate to suppress procancerous polarization while favoring pro-immune, anti-cancerous macrophages?

A growing body of evidence implicates tissue stressors—such as oxidative stress, hypoxia, acidosis, and hemorrhage—in driving TAMs toward immunosuppressive phenotypes characterized by diminished tumoricidal capacity, pro-angiogenic activity, and a propensity to facilitate epithelial-mesenchymal transition (EMT), invasion, and metastasis.^5 6^ Therapeutic interventions, such as immunotherapies and high-dose radiotherapy, exacerbate these stress conditions by inducing cell death and hemorrhage, forcing macrophages to adapt.^3 7^ A primary mediator of stress adaptation is the transcription factor NRF2 (NFE2L2).^8 9^ Under basal conditions, NRF2 is negatively regulated by KEAP1; however, upon oxidative or electrophilic stress, NRF2 stabilizes and translocates to the nucleus, driving a cytoprotective transcriptional response. While NRF2 protects macrophages against oxidative insults, it concurrently impairs their antitumor capacity, diverting them from pro-inflammatory functions to an immunosuppressive state.^10^,^13^ In hemorrhagic tumor areas, for example, TAMs encountering damaged red blood cells and heme upregulate NRF2-responsive genes, biasing them toward procancerous phenotypes.^514^,^17^ Consequently, macrophage-based stress resilience through NRF2 may inadvertently fuel immunosuppressive TAM states, advancing cancer progression and undermining immunotherapies.

Here, we show that immunotherapeutic interventions expand TAM phenotypic diversity along a bifurcated polarization trajectory: immune-activated Cxcl9^+^ macrophages accumulate at the tumor periphery, whereas immunosuppressive Spp1^+^ macrophages localize to perinecrotic regions where NRF2 activity is high. Our work identifies NRF2 as a driver of therapy-resistant, cancer-promoting TAM subsets and uncovers a mechanistic link between stress adaptation and immunotherapy resistance in the TME. We further identify that modifying NRF2 could restore immunogenic macrophage subsets, thereby enhancing immunotherapy outcomes. This is the first comprehensive dissection of how immunotherapy-related necrosis drives an NRF2-centered macrophage program that subverts T-cell infiltration and tumor control.

## Results

### Rebound polarization of monocyte-derived tumor macrophages under repeated anti-CD40 immunotherapy

**Figure 1:**
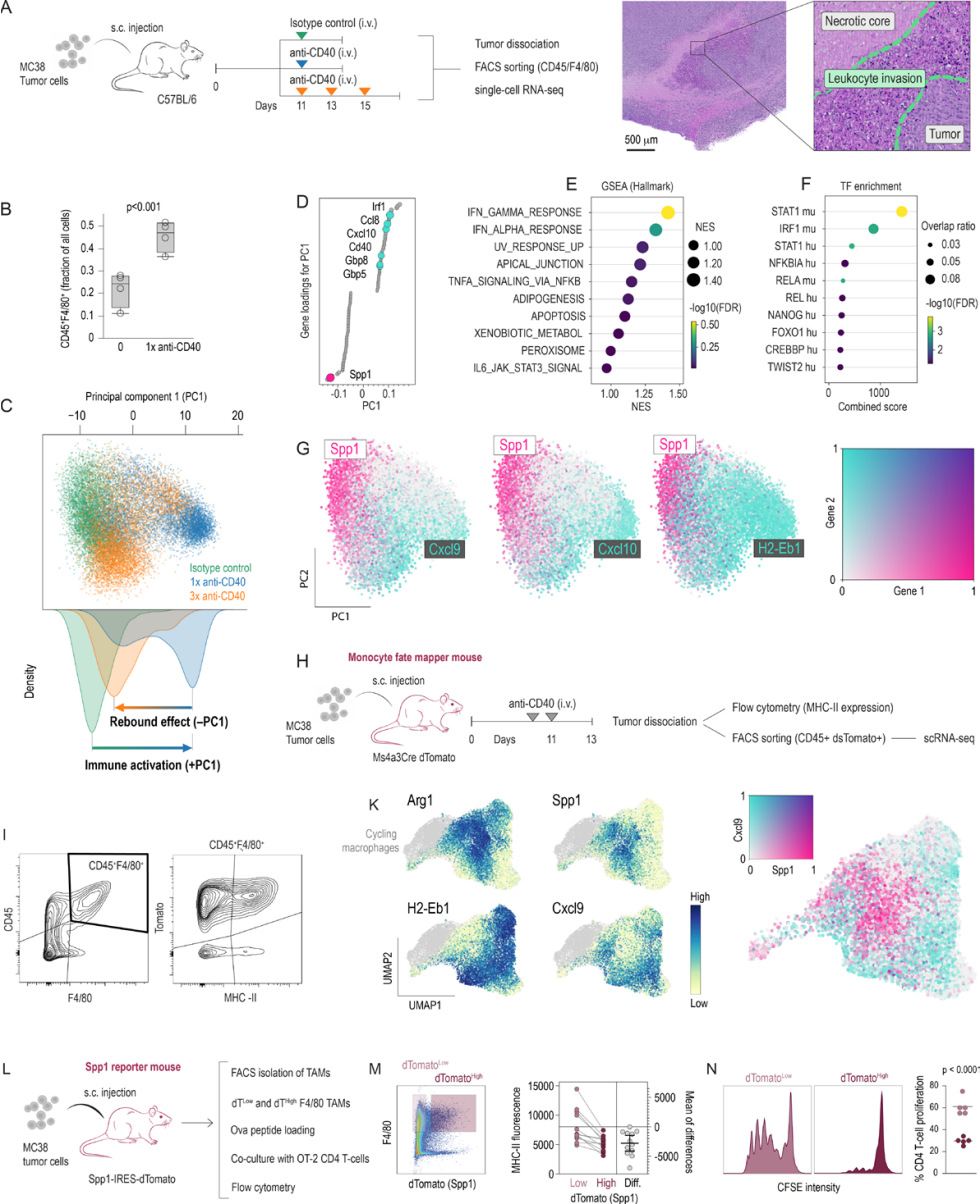
Biphasic polarization of tumor macrophages under anti-CD40 immunotherapy. (**A**) Mice bearing s.c. MC38 tumors were treated intravenously with either isotype control or agonistic anti-CD40 antibodies administered either as a single dose or as a three‑dose regimen. 24 hours after the final injection, tumors were harvested, and CD45^+^ F4/80^+^ cells were FACS-sorted for multiplexed scRNA-seq. Anti-CD40 therapy induces extensive tumor necrosis with patchy leukocyte infiltration as observed in the H&E-stained MC38 tumor section (right). (**B**) Flow cytometry of MC38 tumor single-cell suspensions indicates increased macrophage infiltration following anti-CD40 injection. Bars represent the fraction of CD45^+^F4/80^+^ cells among total live tumor cells (n=3 per group; mean±SD, t-test). (**C**) After demultiplexing and normalization, we selected the macrophage compartment and performed PCA on the scRNA-seq data. The scatter plot of the first two principal components (PC1, PC2) is color-coded by treatment group. Density plots for PC1 highlight that repeated anti-CD40 therapy shifts the macrophage transcriptome back toward the control-like phenotype. (**D**) Gene loadings for PC1: genes are ordered by their contribution (loading) to PC1. High positive loadings indicate genes strongly associated with the positive PC1 direction (eg, Cxcl10), whereas negative loadings highlight genes such as Spp1. (**E**) GSEA: the top PC1-loading genes (positive direction) were subjected to GSEA (Hallmark gene sets). Dots are color-coded and size-coded by −log_10(FDR) and NES, respectively. (**F**) TF over-representation: positive PC1-loading genes were further analyzed for TF-binding motifs. The dot plot displays key TFs (eg, STAT1) enriched among these genes. (**G**) Scatter plots of PC1 versus PC2 are color-coded for Spp1/Cxcl9, Spp1/Cxcl10, or Spp1/H2-Eb1 expression. These data illustrate a polarity between Spp1^+^ immunosuppressive and Cxcl9^+^ /Cxcl10^+^ immunogenic macrophages. (**H**) Monocyte origins: mice bearing MC38 tumors were injected with anti-CD40 twice, followed by tumor harvest. CD45^+^tdTomato^+^ myeloid cells were FACS-sorted for scRNA-seq. The Ms4a3-Cre Rosa26-tdTomato fate-mapping system labels monocytes and their progeny irreversibly. (**I**) Flow cytometry of tumor cell suspension shows that most tumor-associated macrophages (TAMs) in the MC38 model (Ms4a3-Cre fate-mapper) are CD45^+^ F4/80^+^ tdTomato^+^, with subsets differing in MHC class II levels. (**K**) scRNA-seq UMAP of CD45^+^tdTomato^+^ macrophages from Ms4a3-Cre mice treated with anti-CD40. Expression feature plots for Arg1, Spp1, H2-Eb1, and Cxcl9 confirm a bifurcated trajectory to immunosuppressive (Spp1^+^, Arg1^+^) and pro-inflammatory (Cxcl9^+^, H2-Eb1^+^) macrophages. (**L**) MC38 tumors were grown in Spp1-IRES-dTomato mice and treated with anti-CD40 antibody. (**M**) CD45^+^F4/80^+^ TAMs from n=12 tumors were stratified for high versus low dTomato fluorescence and MHC-II expression quantified as mean fluorescence. (**N**) dTomato^high^ and dTomato^low^ CD45^+^F4/80^+^ TAMs from n=5 tumors were FACS-sorted, loaded with OVA^323–339^ peptides, and co-cultured with naive CFSE-labeled CD4^+^ T-cells from OT-II mice. After 72 hours, we found less T-cell proliferation after co-culture with dTomato high versus dTomato low TAMs (paired t-test). CFSE, carboxyfluorescein succinimidyl ester; FACS, fluorescence-activated cell sorting; FDR, false discovery rate; GSEA, Gene Set Enrichment Analysis; MHC, major histocompatibility complex; NES, normalized enrichment score; s.c., subcutaneous; scRNA-seq, single-cell RNA sequencing; TAM, tumor-associated macrophage; TF, transcription factor; UMAP, uniform manifold approximation and projection.

To investigate stress-induced TAM phenotypes on immunotherapy, we treated colon carcinoma MC38 tumor-bearing mice with a single dose of agonistic anti-CD40 or isotype control antibodies on day 11 (1×anti-CD40) or with repeated doses (3×anti-CD40). The repeated-dose protocol induces extensive necrosis with patchy zones of dense leukocyte infiltration in MC38 tumors (figure 1A). Tumors were harvested 24 hours after the last injection. Anti-CD40 injection significantly elevated the number of tumor-infiltrating CD45^+^F4/80^+^ macrophages (figure 1B). We isolated these CD45^+^F4/80^+^ TAMs by fluorescence-activated cell sorting (FACS) for single-cell RNA sequencing (scRNA-seq) and subsequent principal component analysis (PCA) to capture the primary sources of variation across samples (figure 1C,D). The sample distribution along the first principal component (PC1) was consistent with an acute immune activation induced by the single antibody injection protocol, followed by a rebound on triple antibody injection. To identify which genes drive this separation, we examined PC1 loadings, which measure the contribution of each gene to PC1. Genes with high positive loadings (eg, Irf1, Ccl8, Cxcl10, Cd40, Gbp5, Gbp8) characterized the initial anti-CD40 antibody effect, consistent with an IFN–STAT1–driven, immune-activated phenotype (figure 1E,F).^18^ In contrast, Spp1 exhibited a strong negative loading, aligning with the rebound. Spp1 is a marker of immunoresilient and procancerous TAMs.^19^ Therefore, this rebound raised questions about stress signals in the TME driving macrophages toward an immunosuppressive trajectory. Across all three samples, figure 1G highlights the striking polarity along a phenotype gradient from immune-activated Cxcl9^+^ Cxcl10^+^ MHC-II^+^ macrophages towards Spp1^+^ TAMs. While Cxcl9^+^ and Spp1^+^ TAMs define opposite poles along a continuum of polarization, many cells occupy intermediate states that co-express features of both programs.

To confirm that both pro-inflammatory and immunosuppressive macrophage subsets arise from common monocyte precursors, we employed Ms4a3-Cre Rosa26-tdTomato fate-mapping mice (figure 1H).^20^ Flow cytometry of dissociated tumors showed that most F4/80^+^ macrophages were tdTomato^+^, with subpopulations differing in major histocompatibility complex (MHC)-II expression (figure 1I). scRNA-seq of CD45^+^ tdTomato^+^ monocyte progenies supported a bifurcated differentiation trajectory that yields pro-inflammatory, MHC-II-high macrophages (Cxcl9^+^, H2-Eb1^+^), as well as Spp1^+^ Arg1^+^ immunocompromised phenotypes (figure 1K).

Given that repeated anti-CD40 therapy enriches for Spp1^+^ macrophages, we next employed Spp1-IRES-dTomato reporter mice to test whether Spp1 expression directly associates with reduced immunogenic capacity of TAMs (figure 1L). Flow cytometric analysis confirmed that CD45^+^F4/80^+^ TAMs with high Spp1 reporter activity express lower MHC- II (figure 1M) and show impaired OVA^323–339^ antigen presentation to carboxyfluorescein succinimidyl ester (CFSE)-labeled OT-II CD4^+^ T cells, resulting in diminished T-cell proliferation in co-culture (figure 1N). Thus, Spp1^+^ macrophages in rebound-state tumors manifest a hypoimmune phenotype that correlates directly with weakened T-cell priming ability, reinforcing the role of Spp1^high^ TAMs as a marker and driver of immune evasion.

These data suggest that as therapy-induced stress intensifies, macrophages adopt an Spp1^high^ state correlated with diminished MHC-II and suboptimal T-cell priming. This “rebound” phenomenon suggested that region-dependent adaptation could counter the immune-activating signals in stressed tumor microenvironments—an inquiry we pursued by examining the spatial organization of TAMs.

### Spatial zonation of pro-inflammatory and anti-inflammatory tumor macrophages after anti-CD40 and radiation therapy

**Figure 2:**
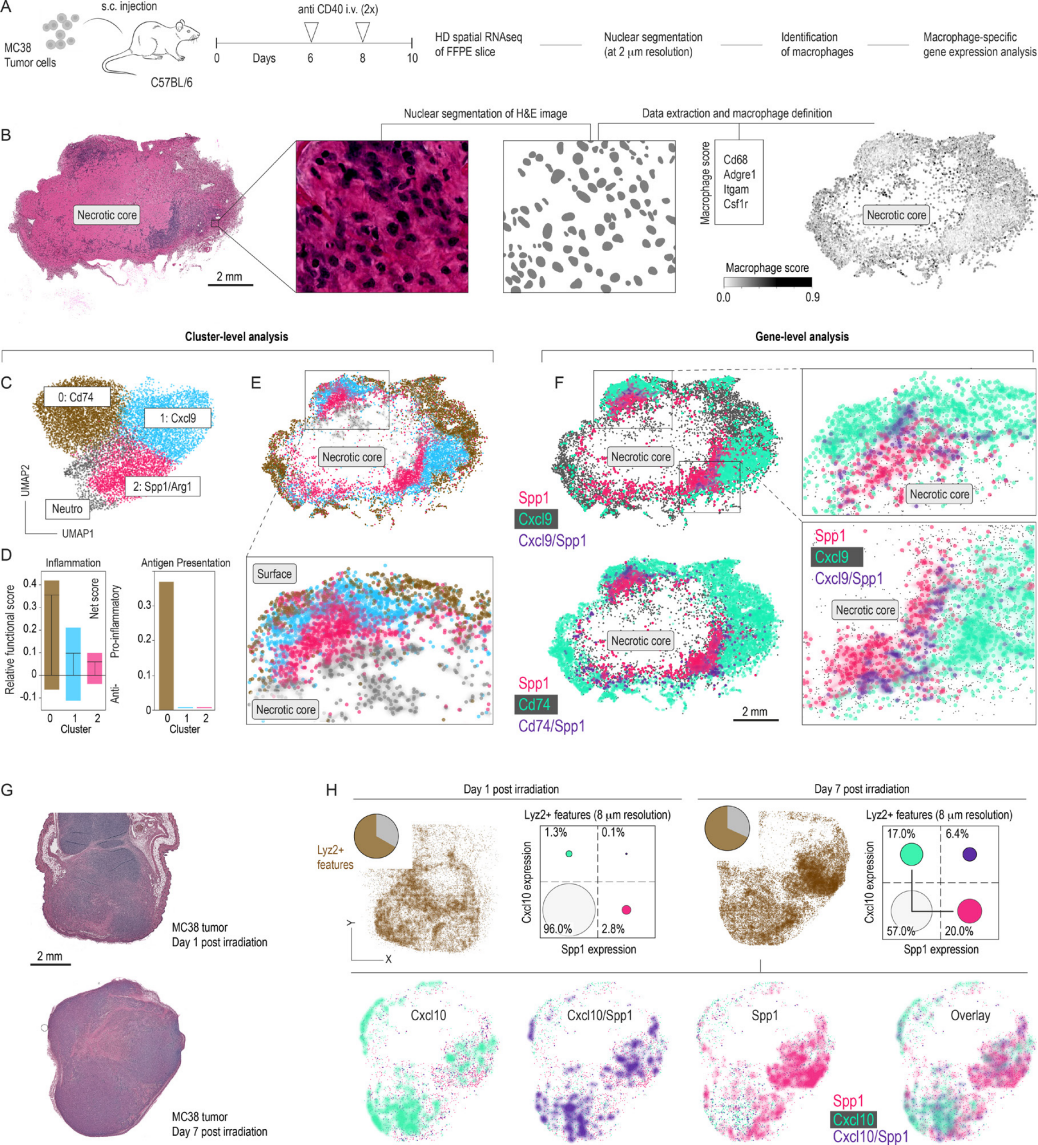
Spatial zonation of heterogeneous TAM phenotypes after anti-CD40 or radiation. (**A**) Mice bearing s.c. MC38 tumors were treated intravenously with agonistic anti-CD40 antibodies on days 6 and 8 before they were harvested on day 10 and processed for HD spatial gene expression analysis. (**B**) H&E-stained MC38 tumor section identifying a necrotic core. A higher-magnification image shows the nuclear segmentation used to achieve single-cell resolution. Macrophages were annotated using a score of marker genes. (**C**) Clustering of macrophage transcriptomes reveals three subsets defined by the marker genes: Cd74, Cxcl9, and Spp1/Arg1. A small cluster (Neutro) was reclassified as neutrophil granulocytes. (**D**) Weighted inflammation and antigen presentation scores per cluster indicate a pro-inflammatory (>0) versus anti-inflammatory (<0) polarization. (**E**) Spatial cluster mapping of these macrophage subsets highlights distinct zonation relative to the necrotic core. Spp1/Arg1 macrophages accumulate peri-necrotically, whereas Cxcl9 and Cd74 macrophages localize toward the tumor periphery. (**F**) Spatial distribution of Spp1^+^, Cxcl9^+^, and Cd74^+^ cells further emphasizes gene expression gradients within the tumor. The spatial zonation depicted here not only maps macrophage diversity but also correlates with regions likely to influence T-cell recruitment and activation. (**G**) MC38-bearing mice were irradiated with 12 Gy of radiation, and tumors were harvested 24 hours or 7 days post-irradiation for HD spatial sequencing. H&E-stained sections of these tumors are shown. (**H**) Spatial RNA-seq analysis of tumor macrophages. The fractions of Lyz2^+^ features are comparable between the two samples when analyzed at 8 μm spot resolution. The comparison of day 1 and 7 post-irradiation samples shows increased phenotype polarization of macrophages along the Spp1-Cxcl10 expression axis; the size of each dot indicates the relative percentage. The 7-day post-irradiation sample was further analyzed at single-nucleus resolution, yielding spatially separated regions occupied by Spp1^+^ and Cxcl10^+^ macrophages. HD, high definition; s.c., subcutaneous; scRNA-seq, single-cell RNA sequencing; TAM, tumor-associated macrophage.

To understand how local tissue factors reinforce these divergent TAM phenotypes, we investigated their spatial distribution in MC38 tumors following two anti-CD40 treatments (on days 6 and 8 post-inoculation) (figure 2A). We performed high-definition spatial transcriptomics at a spatial resolution of ~2 µm on a formalin-fixed, paraffin-embedded tumor section. H&E staining of the analyzed sample revealed dense leukocyte infiltrates and a sizeable necrotic core (figure 2B). After segmenting single nuclei, we identified macrophages guided by canonical markers and extracted the macrophage-specific transcriptome data for subsequent cell-by-cell analysis. Among the four macrophage clusters (figure 2C), the small cluster 3 was reclassified as neutrophil granulocytes after manually inspecting the expressed genes. We classified the differentially expressed genes of the three remaining clusters as pro-inflammatory, anti-inflammatory, or neutral and calculated weighted “inflammation” and “antigen presentation” scores for each cluster (figure 2D). Cluster 0 expressed high levels of MHC-II–associated genes (eg, Cd74), reflecting an antigen-presenting, pro-inflammatory state, whereas cluster 2 predominantly expressed Spp1 and Arg1, typical of anti-inflammatory macrophages. Cluster 1 exhibited moderate pro-inflammatory features, including expression of the T-cell chemoattractant Cxcl9, but lacked prominent antigen presentation.

The spatial mapping of the cluster identities showed a clear partition in macrophage phenotypes (figure 2D). The peripheral zone was rich in macrophages classified as antigen-presenting and pro-inflammatory, while anti-inflammatory macrophages were concentrated close to the necrosis-proximal region (figure 2E). In a gene-level analysis, this pattern was consistent with the accumulation of Cd74^+^ and Cxcl9^+^ macrophages at the tumor edges and Spp1^+^ macrophages clustering toward the necrotic center (figure 2F). Cells co-expressing pro-inflammatory and anti-inflammatory markers (Cxcl9^+^/Cd74^+^ and Spp1^+^) were scattered between the single-positive populations, suggesting a continuous gradient of macrophage states driven by local stress.

The spatial distribution of TAMs, as revealed by our high-definition transcriptomics, not only delineates distinct regional phenotypes but also provides a framework to understand subsequent immune responses. Specifically, the accumulation of pro-inflammatory, antigen-presenting macrophages at the tumor periphery suggests a favorable niche for T-cell activation. In contrast, the concentration of Spp1^+^ macrophages near necrotic zones likely impedes effective T-cell priming and recruitment. This spatial heterogeneity sets the stage for the functional T-cell impairment presented in later sections of this paper.

Because multiple therapeutic modalities can induce necrosis, we analyzed irradiated MC38 tumors to see if they similarly exhibit Spp1^+^– Cxcl9^+^ polarization. Therefore, we conducted a spatial RNA-seq analysis on MC38 tumors 24 hours and 7 days post-irradiation with 12 Gy. Figure 2G shows the H&E-stained slices that were analyzed. As observed under anti-CD40-mediated immunotherapy, we discovered that phenotype heterogeneity among Lyz2^+^ features increased over time (figure 2H), reproducing the bifurcated polarization trajectory along the Spp1–Cxcl9/Cxcl10 axis. High-resolution macrophage profiling similarly confirmed the regional segregation of Cxcl10^+^ (better detected in these samples than Cxcl9) and Spp1^+^ cells.

The convergence of rebound immunosuppression seen with repeated anti-CD40 therapy and this necrosis-proximal enrichment of Spp1^+^ TAMs in both treatments prompted us to explore how necrotic tissue damage-activated pathways, such as NRF2, might underlie macrophage plasticity in these stressed conditions.

### An NRF2-mediated stress response fuels TAM heterogeneity

**Figure 3:**
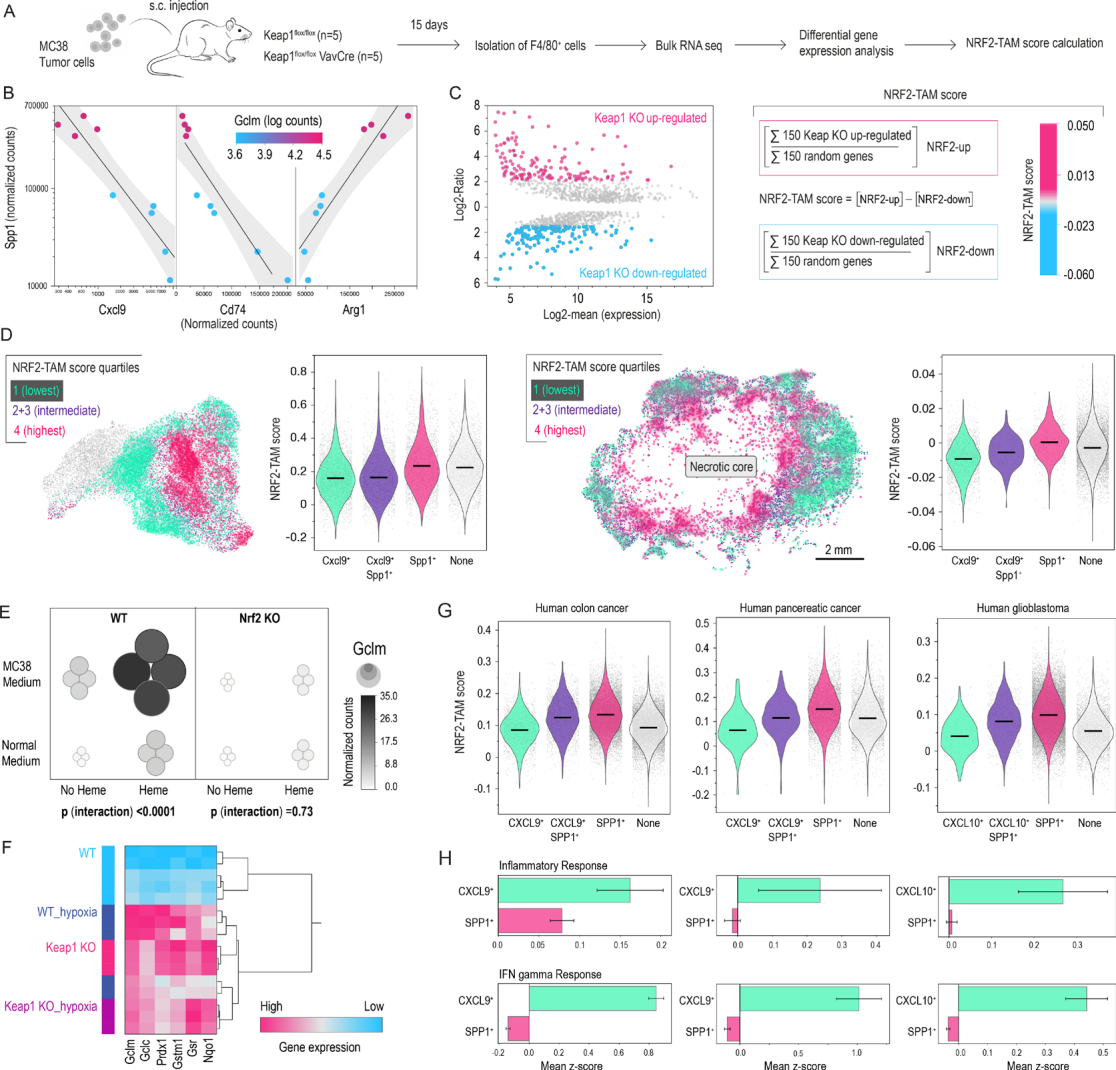
An NRF2-driven transcriptional program defines the immunotherapy-associated TAM dichotomy. (**A**) MC38 tumors grown s.c. in Keap1^flox/flox^VavCre (KO; n=4) and WT (n=5) mice were collected on day 15. TAMs (F4/80+) were purified and analyzed by bulk RNA-seq. (**B**) Correlation analysis of Spp1, Cxcl9, Cd74, and Arg1 expression in Keap1 KO versus WT TAMs, with color-coded Gclm (an NRF2 target). Keap1 loss strongly enhances Gclm expression and shifts TAMs toward an immunosuppressive phenotype (high Spp1/Arg1, low Cxcl9/Cd74). (**C**) MA plot showing log-fold change versus average expression of differentially expressed genes in Keap1 KO versus WT TAMs. The 150 most overexpressed and underexpressed genes define a gene signature used to calculate a score for NRF2-imprinted stress-TAMs. (**D**) NRF2-imprinted stress-TAMs scores were calculated cell-by-cell for the scRNA-seq data in figure 1K (left) and the spatial RNA-seq data in figure 2A–E (right). Features within the highest and lowest score quartiles were highlighted in the UMAP and spatial maps, respectively, mirroring the geography of macrophage polarization states. Violin plots visualize increasing NRF2-imprinted stress-TAM scores in the order of Cxcl9^+^, Cxcl9^+^/Spp1^+^, and Spp1^+^ features (ANOVA with Tukey-Kramer, p<0.001 for all pairwise comparisons). (**E**) BMDMs (WT vs Nrf2 KO) were treated with regular or MC38-conditioned medium±heme. Regression modeling indicated strong synergistic effects (p for interaction heme×MC38 medium) of the two stimuli on Gclm expression in WT but not Nrf2 KO macrophages. Gene expressions were measured by RT-qPCR and normalized to Hprt. Each bubble or dot represents macrophages from one animal. (**F**) Hierarchical clustering of canonical NRF2 target genes in Keap1 or WT BMDMs cultured for 96 hours under normoxia (21% O_2_) or hypoxia (0.2% O_2_). Each row represents an independent bulk-RNA-seq sample (three to five biological replicates per condition). The heat map displays column-wise, normalized log_2_-transformed counts; magenta indicates high gene expression, and blue indicates low gene expression. WT hypoxia and Keap1 KO samples group within the same cluster, suggesting that both conditions strongly enhance the expression of NRF2 target genes. (**G**) Human scRNA-seq reanalysis of colon (341,170 cells, 205 samples), pancreatic (12,200 cells, 20 samples from two studies), and glioblastoma cancers (n=30,864 cells, 31 samples) reveals that SPP1^+^ macrophages have a higher NRF2-imprinted-TAM score, whereas lower scores align with CXCL9^+^/CXCL10^+^/CD74^+^ subsets (ANOVA with Tukey-Kramer post-test, p<0.001 for all pairwise comparisons). (**H**) Inflammatory response and interferon gamma response scores (hallmark gene sets) were calculated for each cell. Bars show the mean±95% CI per subset within each tumor type. CXCL9^+^ and CXCL10^+^ macrophages exhibit significantly higher inflammatory and IFN-γ signatures than SPP1^+^ macrophages in all three cancers. ANOVA, analysis of variance; BMDM, bone marrow-derived macrophage; IFN, interferon; KO, knockout; s.c., subcutaneous; scRNA-seq, single-cell RNA sequencing; RNA-seq, RNA sequencing; RT-qPCR, reverse transcription quantitative real-time; TAM, tumor-associated macrophage; UMAP, uniform manifold approximation and projection; WT, wild-type.

Tissue ischemia, necrosis, and injury-associated toxins such as heme are typically sensed by macrophage NRF2, triggering an adaptive response.^1215 21^,^23^ Therefore, we hypothesized that NRF2 might orchestrate the shift toward Spp1^+^ TAMs. To characterize a prototypical phenotype of NRF2-driven TAMs, we purified macrophages from MC38 tumors grown in Keap1^flox/flox^ VavCre mice (n=4) and wild-type (WT) controls (n=5) (figure 3A). In these conditional Keap1 knockout (KO) mice, NRF2 is constitutively activated in hematopoietic cells. Bulk RNA-seq of F4/80^+^ TAMs showed widespread transcriptional reprogramming consistent with persistent NRF2 activity, including elevated Gclm (a canonical NRF2 target). Importantly, the NRF2-activated TAMs reflected hallmark features of our necrosis-associated macrophages with increased Spp1 and Arg1 expression, negatively correlated with Cxcl9 and Cd74 (figure 3B).

We called these TAMs “NRF2-imprinted stress-TAMs” and established a signature of NRF2-imprinted macrophages based on the top 150 most differentially expressed genes (upregulated and downregulated) between Keap1 KO and WT TAMs (figure 3C). This score reflects the downstream phenotypic footprint of NRF2 activation during differentiation rather than instantaneous transcription factor activity. We have used this score to classify the macrophages identified in our MC38 tumor TAM fate mapping study (figure 1K) into NRF2-imprinted TAM score quantiles. The Uniform Manifold Approximation and Projection (UMAP) in figure 3D reveals that the highest quartile scores overlap with Spp1^+^ macrophages, and across the entire data set, Spp1^+^ macrophages had higher NRF2-imprinted TAM score than Cxcl9^+^ cells. In another analysis, we projected the cell-by-cell calculated NRF2-imprinted TAM scores onto the spatial transcriptomics dataset analyzed in figure 2. This revealed a spatial organization with the highest NRF2-imprinted TAM scores close to the necrotic core, again, overlapping strongly with Spp1^+^ macrophages (figure 3D). Also, in this case, cells expressing Spp1 matched high NRF2-imprinted TAM scores, whereas Cxcl9^+^ macrophages had lower scores.

To experimentally establish that NRF2 activation occurs in the tumor tissue-stress context, we turned to an in vitro system. First, bone marrow-derived macrophages (BMDMs) from Nrf2 KO and WT mice were exposed to MC38-conditioned medium and the necrosis-toxin heme, which synergistically induced the NRF2 target gene Gclm in WT but not Nrf2 KO BMDMs. Second, we cultured WT and Keap1 KO BMDMs for 96 hours in hypoxia (0.2% O_₂_). Hypoxia strongly elevated the expression of canonical NRF2 targets in WT cells, clustering with normoxic Keap1-deficient macrophages in the NRF2 target-gene space. These observations suggest that archetypal TME stresses broadly activate NRF2 in macrophages (figure 3F).

To generalize our observation that the dichotomous Cxcl9^+^ and Spp1^+^ TAM spectrum reflects an NRF2 activation gradient, we reanalyzed publicly available scRNA-seq data from human colorectal and pancreatic cancers as well as glioblastoma (figure 3G).^24^ Consistent with our observations in the mouse model, we identified the same pattern across these human cancer types: CXCL9^+^ macrophages with broadly pro-inflammatory and interferon (IFN) gamma-activated gene expression profiles (figure 3H) exhibited the lowest NRF2-imprinted TAM scores; anti-inflammatory SPP1^+^ macrophages displayed the highest scores, and double-positive cells showed intermediate scores.

Collectively, these findings establish a model in which microenvironmental stress drives the transition of macrophage phenotypes from pro-inflammatory to anti-inflammatory states. This macrophage phenotype shift may influence macrophage immune functions, their interactions with tumor cells, and therapeutic responses.

### NRF2 drives anti-inflammatory stress macrophages

**Figure 4:**
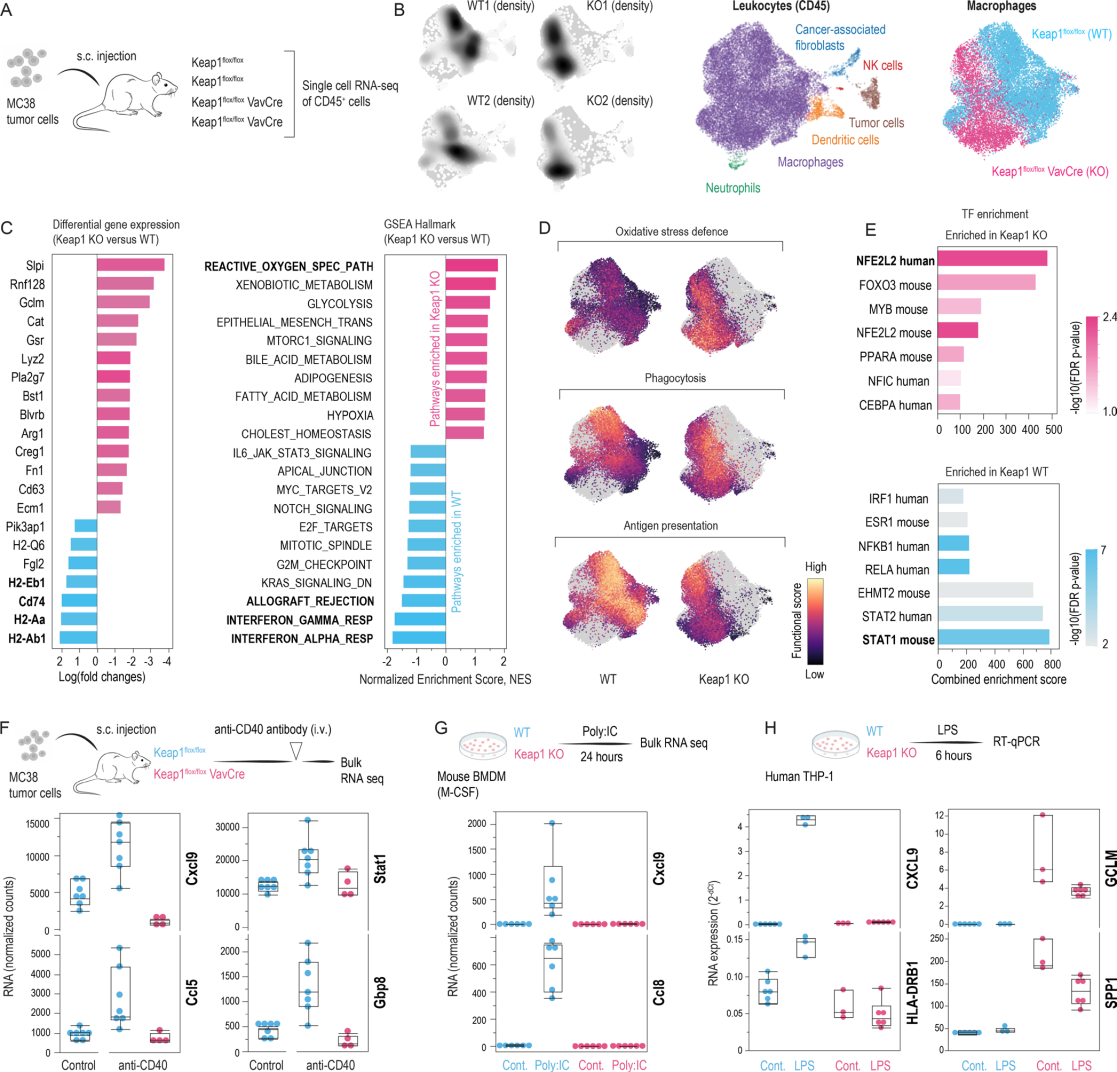
NRF2-imprinted stress-TAM identity and responsiveness to immunostimulatory cues in MC38 tumors. (**A**) MC38 tumors grown s.c. in Keap1^flox/flox^ VavCre (KO; n=2) or WT (n=2) mice were harvested on day 15. CD45^+^ leukocytes were analyzed by scRNA-seq to reveal NRF2-driven macrophage programs. (**B**) Integrated UMAP with sample-wise cell density projections showing genotype-dependent transcriptomic shifts, with macrophages as the most abundant leukocyte subset. (**C**) Differential gene expression and hallmark GSEA identify key pathways (eg, oxidative stress defense) elevated in Keap1 KO TAMs. (**D**) Functional score maps indicate increased oxidative stress defence but reduced antigen presentation in Keap1 KO versus WT TAMs, with comparable phagocytic capacity. (**E**) Transcription factor over-representation analysis reveals NFE2L2 (NRF2) as the top-activated and STAT1 as the repressed factor in KO TAMs, suggesting an anti-inflammatory, immunodeficient phenotype. (**F**) Expression (normalized counts) of selected genes in WT versus Keap1 KO TAMs after anti-CD40 treatment shows that NRF2 confers resistance to macrophage-activating immunotherapy. (**G**) WT versus Keap1 KO BMDMs treated with poly(I:C) confirm that NRF2 constrains the macrophage response to inflammatory stimuli. (**H**) THP-1-derived human macrophages (KEAP1 WT and KO) were stimulated with LPS. After 6 hours HPRT-normalized expression of CXCL9, HLA-DRB1, SPP1 and GCLM was quantified by RT-qPCR (ANOVA with Tukey-Kramer post-test, CXCL9 WT LPS vs KEAP1 KO LPS p<0.0001, HLA-DRB1 WT LPS vs KEAP1 KO LPS p<0.0001, SPP1 control WT vs control KEAP1 KO p<0.0001, GCLM control WT vs control KEAP1 KO p<0.0001; n=3–6). ANOVA, analysis of variance; BMDM, bone marrow-derived macrophage; FDR, false discovery rate; GSEA, Gene Set Enrichment Analysis; i.v., intravenous; KO, knockout; LPS, lipopolysaccharide; M-CSF, macrophage colony-stimulating factor; NK, natural killer; s.c., subcutaneous; scRNA-seq, single-cell RNA sequencing; RNA-seq, RNA sequencing; RT-qPCR, reverse transcription quantitative real-time; TAM, tumor-associated macrophage; TF, transcription factor; WT, wild-type.

Next, we evaluated the function of NRF2-imprinted stress-TAMs and how they respond to immunostimulatory cues by performing scRNA-seq of CD45^+^ tumor-infiltrating leukocytes from Keap1^flox/flox^ VavCre and WT mice (n=2 per group) (figure 4A). As shown in figure 4B, an integrated UMAP of scRNA-seq data demonstrates that macrophages, which were the most abundant leukocyte population, cluster distinctly in WT versus Keap1 KO mice, indicating a genotype-dependent shift in their transcriptome. Differential expression, Gene Set Enrichment Analysis (GSEA), and cell-by-cell functional scoring using published gene sets for specific macrophage functions^25^ indicated a pronounced upregulation of oxidative-stress defense and metabolic adaptation in Keap1 KO macrophages. This was accompanied by reduced MHC-II expression, impaired antigen presentation capacity, and suppressed IFN-response pathways, which are central signaling pathways of antitumor macrophage reprogramming (figure 4C,D). Despite significant shifts in oxidative stress defense and antigen presentation, key macrophage-defining functions such as phagocytic capacity remained comparable between WT and Keap1 KO TAMs. Transcription factor motif analysis further identified NRF2 as a top-activated factor and STAT1 as a repressed factor in Keap1 KO TAMs (figure 4E).

In an anti-CD40 therapy context, F4/80^+^ macrophages from WT tumors responded with robust induction of pro-inflammatory genes (Cxcl9, Ccl5, and IFN-responsive genes). However, Keap1 KO TAMs were largely refractory, indicating that NRF2 restricts macrophage plasticity under immunostimulatory conditions (figure 4F). Parallel in vitro assays with poly(I:C)-stimulated BMDMs confirmed the same phenomenon, demonstrating that NRF2 activation restricts macrophage responses to inflammatory stimuli (figure 4G). To assess conservation in a human model, we generated a KEAP1 knockout THP-1 cell line using CRISPR/Cas9 gene editing and differentiated it with phorbol 12-myristate 13-acetate (PMA) into macrophages. On lipopolysaccharide (LPS) stimulation, KEAP1 KO macrophages exhibited profoundly suppressed CXCL9 expression compared with WT cells. GCLM was enhanced in the KEAP1 KO cell line, consistent with constitutive NRF2 activation (figure 4H).

These findings further emphasize the role of NRF2 in driving an immune-resilient macrophage phenotype that resists reprogramming by therapeutic interventions. Given that TAM-mediated antigen presentation is crucial for T-cell priming, we next examined whether NRF2-imprinted stress-TAMs influence T-cell proliferation and immunity.

### NRF2-activated TAMs undermine T-cell-based tumor immunity

**Figure 5:**
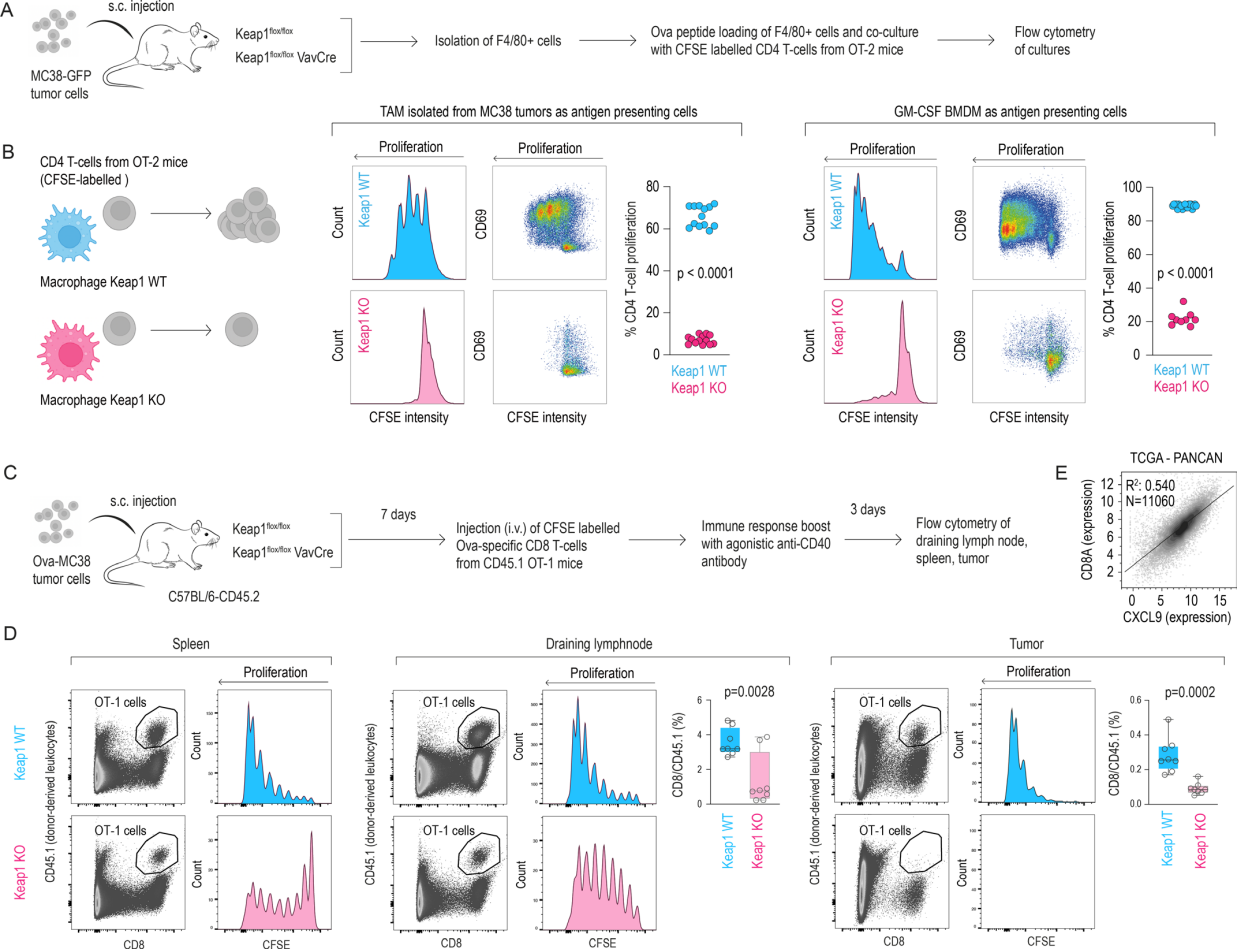
NRF2 activation in TAMs impairs antigen presentation and reduces T-cell responses. (**A**) Ex vivo assay: BMDMs or MC38 tumor-derived CD45^+^F4/80^+^ TAMs from Keap1^flox/flox^ VavCre versus WT mice were used as antigen-presenting cells after loading with OVA^323–339^ peptides. Co-cultures were performed with naive CFSE-labeled OT-II CD4^+^ T-cells. T-cell activation and proliferation (CFSE dilution, CD69 upregulation) were assessed by flow cytometry. (**B**) T-cells co-cultured with WT TAMs proliferate robustly, while those with Keap1 KO TAMs show reduced CD69 expression and limited proliferation. Similar results were observed using in vitro-differentiated WT versus Keap1 KO BMDMs. (**C**) In vivo assay: OVA-expressing MC38 (OVA-MC38) cells were implanted s.c. into WT or Keap1 KO mice. On day 7, OT-I CD8^+^ T-cells were injected i.v., followed by agonistic anti-CD40 antibody. T-cell proliferation and activation in the spleen, draining lymph node, and tumor were measured by flow cytometry. CD8^+^ T-cells were CFSE-labeled and congenic CD45.1 for proliferation and donor tracking, respectively. (**D**) CFSE dilution profiles show reduced OT-I T-cell expansion in conditional Keap1 KO hosts. Consequently, donor-derived CD8^+^ T-cells are profoundly reduced in draining lymph nodes and primary tumors (n=7–8, mean±SD, t-test). (**E**) In the TCGA PANCAN dataset (n=11,060), high CXCL9 expression strongly correlates with increased CD8^+^ T-cell content. This suggests decreased CXCL9 expression in Keap1 KO TAMs contributes to lower CD8^+^ T-cell infiltration. CFSE, carboxyfluorescein succinimidyl ester; GFP, green fluorescent protein; GM-CSF, granulocyte/macrophage colony stimulating factor; i.v., intravenous; KO, knockout; s.c., subcutaneous; TAM, tumor-associated macrophage; TCGA PANCAN, the cancer genome atlas pan-cancer; WT, wild-type.

We examined the functional consequences of NRF2-imprinted stress-TAM polarization for antigen-specific T-cell priming, using an antigen presentation assay with OVA^323–339^-loaded macrophages co-cultured with CFSE-labeled OT-II (CD4^+^) T-cells (figure 5A). Macrophages used in the assay were either CD45^+^ F4/80^+^ TAMs isolated from MC38 tumors or in vitro cultured BMDMs. Flow cytometry after 72 hours showed that T-cells co-cultured with WT macrophages underwent robust proliferation and upregulated the activation marker CD69. In contrast, T-cells co-cultured with Keap1 KO macrophages showed little to no CFSE dilution or CD69 induction (figure 5B). We established an adoptive transfer set-up to confirm this defect in vivo (figure 5C). We implanted OVA-expressing MC38 cells into WT or Keap1^flox/flox^ VavCre hosts and transferred CFSE-labeled CD8^+^ T-cells from CD45.1 OT-I mice on day 7, followed by agonistic anti-CD40 antibody to boost antigen presentation. Flow cytometry analysis revealed a significant reduction in donor-derived CD45.1 CD8^+^ T-cells in the spleen and tumor-draining lymph nodes of VavCre Keap1^flox/flox^ mice, with almost no specific T-cells detected within their tumors (figure 5D). Further analysis using CFSE-labeled adoptive T-cells demonstrated markedly attenuated proliferation profiles in VavCre Keap1^flox/flox^ mice compared with WT controls. The near absence of OT-I T-cells in VavCre Keap1^flox/flox^ tumors likely reflects a combination of poor antigen presentation and diminished Cxcl9/Cxcl10-driven T-cell recruitment. This assumption is supported by the observation that across the 11,060 tumor samples in the The Cancer Genome Atlas Pan-Cancer (TCGA PANCAN) database (https://www.cancer.gov/tcga), CXCL9 expression predicts better CD8^+^ T-cell infiltration (figure 5E), and NRF2 profoundly suppresses this chemokine.

These findings indicate that the emergence of NRF2-imprinted stress-TAMs severely compromises macrophage-mediated immune functions, impairing antigen presentation and restraining T-cell-mediated immunity. This macrophage NRF2-driven immune suppression highlights an overarching immunosuppressive program by which TAMs contribute to immune evasion. We next investigated whether NRF2-imprinted TAMs not only impair T-cell responses but also actively reshape tumor cell behavior and the broader TME.

### Modeling anti-inflammatory and tumor-promoting functions of NRF2-driven macrophages in vitro

**Figure 6:**
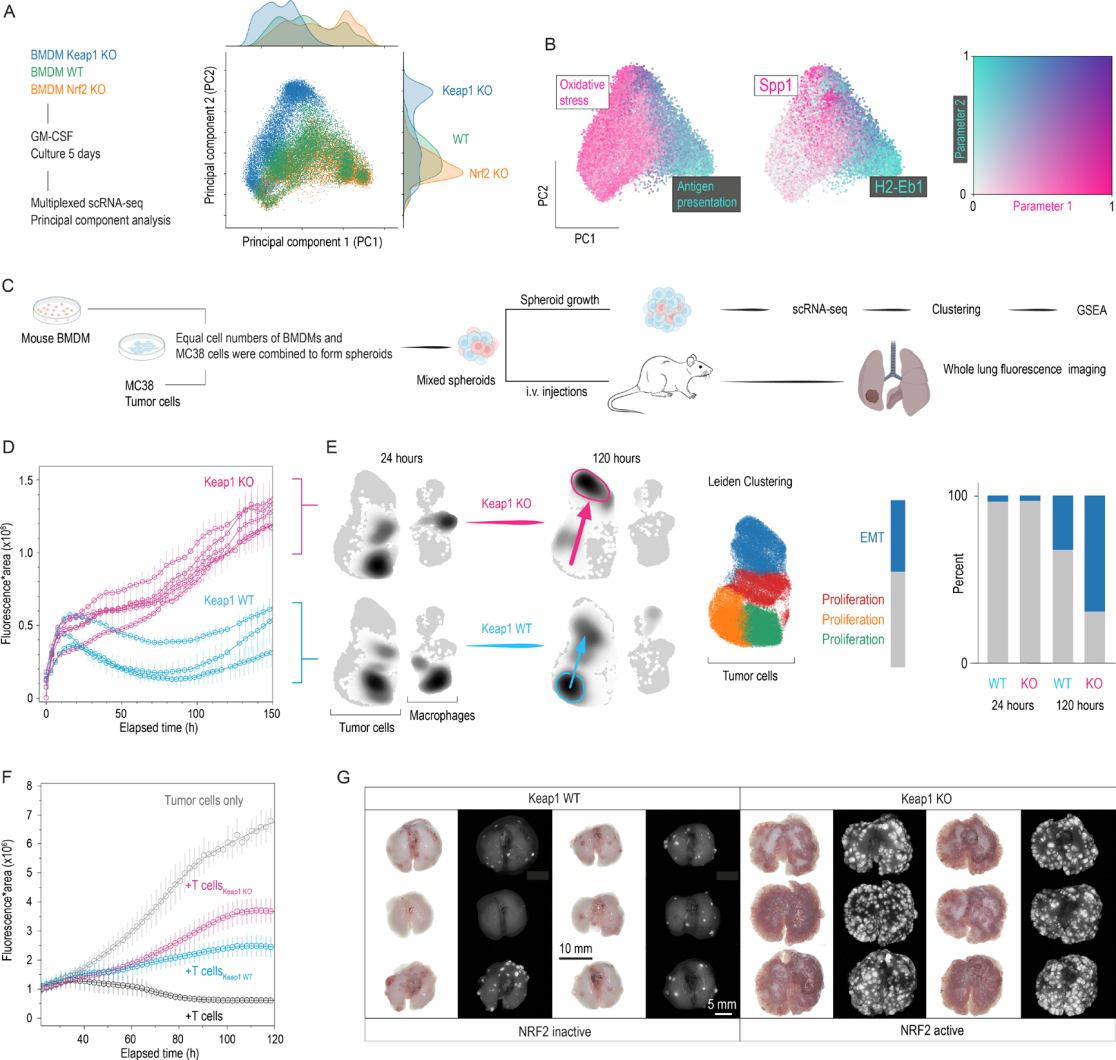
NRF2-driven TAMs promote tumor cell growth, EMT, and attenuate T-cell-mediated killing. (**A**) Multiplexed scRNA-seq of GM-CSF-supported BM macrophages (WT, Nrf2 KO, Keap1 KO). The scatter plot of the first two principal components (PC1 and PC2) is color-coded by treatment group. Density plots for PC1 and PC2 highlight the divergent phenotype between Nrf2 KO, Keap1 KO and WT BM cells. (**B**) Scatter plots of PC1 versus PC2 are color-coded for macrophage function scores and Spp1/H2-Eb1 expression. These data illustrate how NRF2 ablation skews BM cells from Spp1^+^ immunosuppressive towards antigen-presenting macrophages, while NRF2 activation promotes immunosuppression and loss of anti-presentation function. (**C**) Spheroid co-culture model: equal numbers of WT or Keap1 KO BMDMs were mixed with GFP-MC38 cells in ultra-low-attachment plates. Spheroid size, metastatic capacity, and T-cell killing inhibition were assessed. (**D**) Live-cell microscopy tracks spheroid size (area×GFP intensity). Spheroids with Keap1 KO BMDMs grow faster than WT BMDM spheroids. Data as mean±SE (n=4 mice/genotype, 8–10 replicates each). (**E**) Multiplexed scRNA-seq at 24 hours and 120 hours post-spheroid formation. Cell density projections on the integrated UMAP show that the cancer cell transcriptomes are very similar between genotypes despite persistent genotype-dependent variations in the macrophage transcriptome. At 120 hours, cancer cell densities undergo a marked shift, which is much more pronounced in the spheroids containing Keap1 KO macrophages. We performed Leiden clustering of the integrated data and analyzed differentially expressed genes by GSEA using the hallmark gene database. This defined that cancer cells in spheroids with WT macrophages remain mainly proliferative. In contrast, cancer cells in spheroids containing Keap1 KO macrophages progress toward an EMT state (blue cluster). The bar chart (right) quantifies the proportion of cancer cells in proliferative and EMT states. (**F**) T-cell killing assay: activated OT-I CD8^+^ T-cells were kept in regular medium or preconditioned in medium from spheroids containing either WT or Keap1 KO BMDMs, then added to GFP-OVA-MC38 monolayers. Live-cell microscopy tracked the decay of GFP fluorescence relative to the baseline growth dynamics of tumor cells. Spheroid-conditioned medium universally impairs T-cell killing, but the effect is more pronounced in Keap1 KO spheroid medium. Data represent the mean fluorescence intensity (±95% CI) of 8–10 replicates. (**G**) In vivo metastasis assay: lungs harvested 3 weeks after i.v. injection of ~750 spheroids into immunodeficient mice reveal that Keap1 KO-macrophage spheroids produce more extensive metastatic disease compared with WT spheroids. BM, bone marrow; BMDM, bone marrow-derived macrophage; EMT, epithelial-mesenchymal transition; GFP, green fluorescent protein; GM-CSF, granulocyte/macrophage colony stimulating factor; GSEA, Gene Set Enrichment Analysis; i.v., intravenous; KO, knockout; scRNA-seq, single-cell RNA sequencing; RNA-seq, RNA sequencing; TAM, tumor-associated macrophage; UMAP, uniform manifold approximation and projection; WT, wild-type.

To determine whether NRF2-mediated anti-inflammatory differentiation and its downstream consequences on tumor immunology are intrinsic macrophage processes, we conducted a series of in vitro studies. First, we performed scRNA-seq studies of WT, Keap1 KO, and Nrf2 KO BMDMs after short-term culture with granulocyte/macrophage colony stimulating factor (GM-CSF) for 5 days to capture a broad spectrum of differentiation and polarization states. PCA analysis visualizes the significant differences in gene expression patterns between the three genotypes (figure 6A, online supplemental figure 1A). Cell density projections along PC1 and PC2 demonstrated that Nrf2 KO cells were skewed towards MHC-II^high^ macrophages with strong antigen presentation functions. In contrast, the peak density of Keap1 KO cells was shifted away from MHC-II towards Spp1^high^ macrophages with enhanced oxidative stress functions, reinforcing the dose-dependent anti-inflammatory push provided by active NRF2 (figure 6B).

Next, we employed three-dimensional spheroid co-cultures to explore the paracrine and direct cell-contact effects of NRF2-driven mouse or human THP-1-derived macrophages on MC38, TC-1 or HT-29 tumor growth (figure 6C, online supplemental figure 1B). We seeded equal numbers of tumor cells and macrophages (WT or Keap1 KO) in ultra-low-adherence plates to form multicellular spheroids. Live-cell imaging showed that spheroids containing NRF2-imprinted macrophages reached significantly larger sizes over time compared with spheroids with WT macrophages (figure 6D, online supplemental figure 1B). With multiplexed scRNA-seq of MC38 spheroids collected at 24 and 120 hours post-formation, we defined the directed effects of NRF2-imprinted stress-TAMs on tumor cells (figure 6E). UMAP projections of the four samples visualize that the macrophage genotype did not affect tumor cell gene expression within the first 24 hours despite persistent NRF2-driven macrophage phenotypic diversity. However, by 120 hours, tumor cell transcriptomes diverged markedly, as visualized by an unequal shift in cell densities (arrows). This transcriptome shift was much more pronounced in the spheroids containing Keap1 KO macrophages. Leiden clustering and subsequent cluster-based GSEA of the tumor cell transcriptome revealed that while most tumor cells co-cultured with WT macrophages remained in a proliferative state, those co-cultured with Keap1 KO macrophages transitioned into an EMT state, indicative of high metastatic potential.^26^

We further assessed whether the secretome of the macrophage-tumor spheroids may affect T-cell-mediated tumor killing (figure 6F). Before measuring how activated OT-I CD8^+^ T-cells kill their OVA-MC38 target cells, we exposed them to culture medium conditioned by cancer spheroids harboring WT or Keap1 KO macrophages. MC38 proliferation and T-cell-mediated killing were recorded with an Incucyte live cell microscope. T-cells exposed to supernatants from spheroids hosting macrophages showed markedly reduced tumor cell killing, with the effect more pronounced for spheroids containing Keap1 KO macrophages. This finding indicates that NRF2-imprinted stress-TAMs create an immunosuppressive microenvironment that blunts T-cell-mediated tumor lysis (figure 6F).

Since EMT often correlates with metastatic potential,^26^ we next asked whether NRF2-imprinted stress-TAMs influence metastasis in vivo. We intravenously injected approximately 750 macrophage-tumor cell spheroids into immunodeficient mice to assess this. After 3 weeks, we evaluated pulmonary metastases via green fluorescent protein (GFP) fluorescence imaging of the lungs (figure 6G). Mice injected with spheroids containing Keap1 KO macrophages displayed significantly more extensive pulmonary metastases than those with WT macrophages.

Together, these findings demonstrate that in vitro-generated NRF2-imprinted stress-TAMs adopt an anti-inflammatory phenotype, establish an immunosuppressive microenvironment, drive EMT of cancer cells, and suppress T-cell-mediated tumor cell killing, collectively contributing to metastasis and cancer progression. Having confirmed that NRF2-imprinted macrophages potentiate metastasis in a reductionist setting, we next asked whether myeloid NRF2 similarly accelerates tumor progression during spontaneous, therapy-naïve carcinogenesis.

### Macrophage-intrinsic NRF2 accelerates tumor growth in a therapy-naïve setting

**Figure 7:**
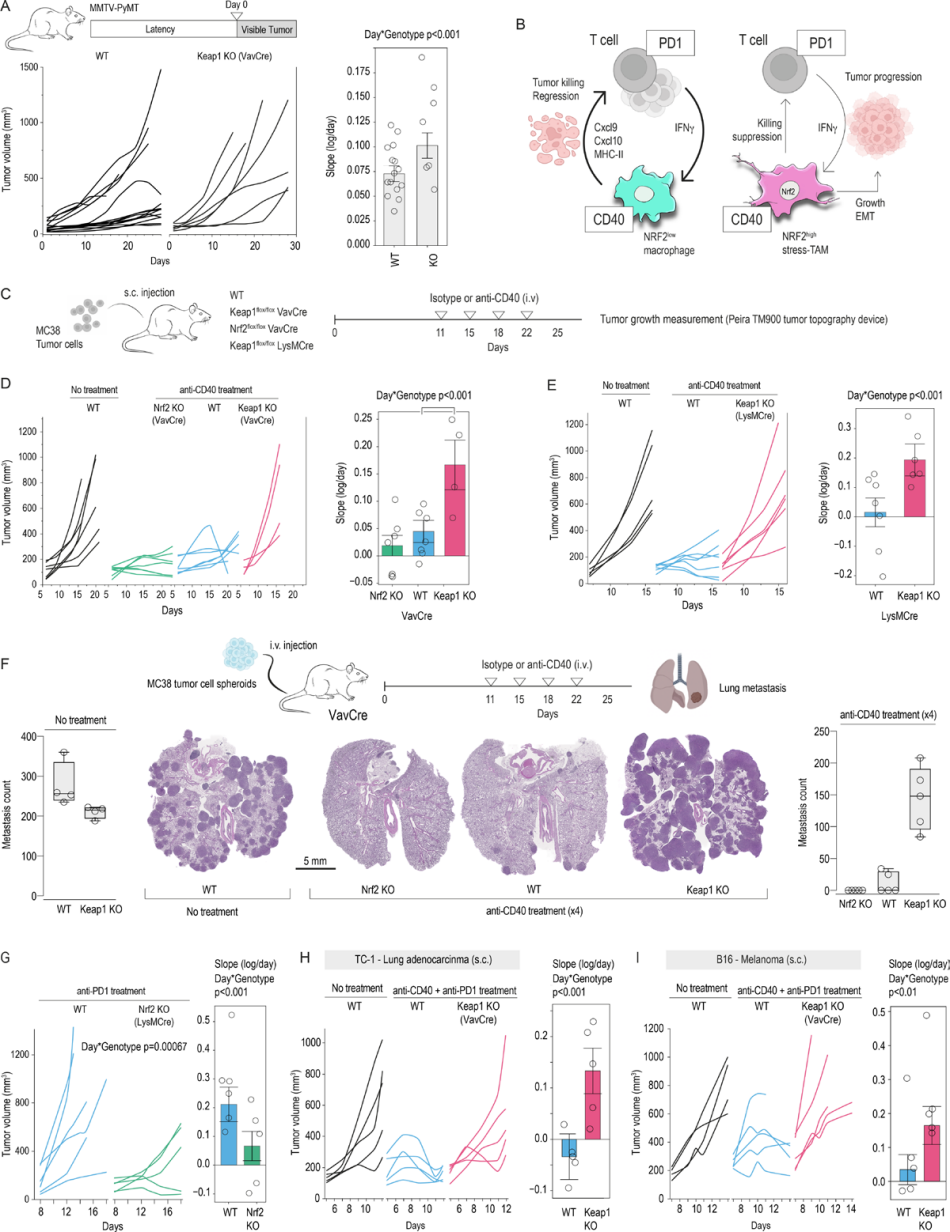
NRF2 in TAMs modulates tumor growth and the efficacy of macrophage-directed immunotherapy. (**A**) Breast cancer growth was measured by digital 3D topography in WT and conditional Keap1 KO MMTV-PyMT females by an investigator blinded for genotypes. Tumors were allowed to grow until they reached 1,000 mm³ or until skin necrosis was imminent. The average latency to first palpable tumor was 125 days. For each animal, day 0 indicates first tumor appearance. A mixed-effects model with day and genotype as fixed effects and animal ID as random effects on log-transformed volumes reveals a significant genotype×day interaction (p=0.0003), indicating significantly enhanced growth rates of tumors in Keap1 KO animals. (**B**) Proposed mechanistic model: under anti‐CD40 or anti‐PD-1 treatments, NRF2-imprinted “stress‐TAMs” subvert tumor clearance, whereas reducing NRF2 activity preserves macrophage/T‐cell functional interactions and enhances tumor regression. (**C**) MC38 tumor-bearing mice were repeatedly treated with either isotype control or agonistic anti-CD40 antibodies i.v. Tumor volumes were measured by 3D topography. (**D**) Growth curves of MC38 tumors in WT and conditional Keap1 and Nrf2 KO mice. Mixed-effect modeling of log-transformed tumor volumes with time and genotype as fixed effects and individual mice as random effects reveals significantly reduced growth slopes in Nrf2 KO and increased slopes in Keap1 KO versus WT. UDL and LDL are upper/lower decision limits at α=0.05. (**E**) MC38 tumors in Keap1^flox/flox^ LysMCre mice also fail to respond to anti-CD40 immunotherapy, underscoring the importance of NRF2 signaling in macrophages. (**F**) Pulmonary metastases were induced by i.v. injection of ~750 MC38 spheroids and analyzed 3 weeks later by histology (H&E staining) and manually counting metastases. Left: without treatment, the disease burden was similar between WT and conditional Keap1 KO mice. Right: anti-CD40 therapy fails to control tumor spread in conditional Keap1 KO mice. Each dot represents one mouse (n=4–5 per genotype, mean±SD, ANOVA with Tukey-Kramer post-test). (**G**) WT and macrophage-specific Nrf2 KO mice were treated with anti‐PD-1 antibodies on days 9, 13, and 16 after subcutaneous MC38 inoculation. Individual tumor volumes show markedly reduced growth in Nrf2 KO. Genotype×day interaction (p=0.0006) indicates significantly lower growth rates of tumors in Nrf2 KO animals. (**H**) WT and conditional Keap1 KO mice were treated with anti-CD40+anti‐PD-1 antibodies on days 7, 10, and 13 after subcutaneous TC-1 inoculation. Immunotherapy fails to control tumor growth in conditional Keap1 KO mice, as indicated by significantly enhanced growth rates in immunotherapy-treated Keap1 KO compared with WT animals (p=<0.001). (**I**) WT and conditional Keap1 KO mice were treated with anti-CD40+anti‐PD-1 antibodies on days 7, 9, and 11 after subcutaneous B16 inoculation. Immunotherapy fails to control tumor growth in conditional Keap1 KO mice, as indicated by significantly enhanced growth rates in immunotherapy-treated Keap1 KO compared with WT animals (p=<0.01). ANOVA, analysis of variance; EMT, epithelial-mesenchymal transition; IFN, interferon; i.v., intravenous; KO, knockout; MHC, major histocompatibility complex; PD-1, programmed cell death protein-1; s.c., subcutaneous; TAM, tumor-associated macrophage; WT, wild-type; 3D, three-dimensional.

To determine whether leukocyte NRF2 promotes tumor progression in the absence of therapeutic stress, we crossed Keap1^flox/flox^ VavCre mice with the spontaneous MMTV-PyMT breast cancer model, a setting in which endogenous immune pressure restrains lesion outgrowth on the C57BL/6 background.^27^ Tumor latency was identical between genotypes (WT 127±25; KO 123.4±31 days), but once palpable, lesions in conditional Keap1 KO hosts expanded significantly faster than those in WT controls (genotype×day p=0.0003; figure 7A). Whereas 9 of 15 WT tumors entered a prolonged plateau phase, all tumors in Keap1^flox/flox^ VavCre mice displayed uninterrupted growth. Thus, constitutive NRF2 activation accelerates tumor progression in a therapy-naïve context, underscoring the broad protumor capacity of the NRF2–TAM axis.

### NRF2 targeting enhances immunotherapy efficacy

Macrophages form a central nexus in a regulatory loop that can promote tumor regression through direct cytotoxicity or T-cell engagement. This loop can be activated by macrophage‐directed (anti‐CD40) and T‐cell engaging (anti‐programmed cell death protein-1 (PD-1)) immunotherapies (figure 7B). We conducted a series of studies to assess our hypothesis that NRF2-imprinted stress-TAM phenotype shifting could undermine this immunotherapeutic paradigm.

Initially, we explored agonistic anti-CD40 therapy, which stimulates antigen presentation and pro-inflammatory cytokine production in macrophages.^18 28^ First, we validated the requirement of macrophages in our model by using mice lacking CD40 on macrophages (Cd40^flox/flox^ LysMCre). Compared with WT mice, these conditional KOs failed to respond effectively to anti-CD40, exhibiting larger tumors with reduced necrosis (online supplemental figure 2).

Next, we monitored tumor growth in MC38-bearing mice that were repeatedly treated with anti-CD40 (figure 7C). Animals included WT, Keap1^flox/flox^ VavCre (NRF2-imprinted), and Nrf2^flox/flox^ VavCre (NRF2-null) cohorts. Mixed-effects modeling of tumor volumes over time showed that conditional Keap1 KO mice were largely resistant to therapy, whereas conditional Nrf2 KO mice displayed a trend towards improved tumor control (figure 7D). Similar results were observed with MC38 tumors in Keap1^flox/flox^ LysMCre mice, underscoring that macrophage-specific NRF2 activation impairs the therapeutic effect of anti-CD40 (figure 7E). Considering that the subcutaneous MC38 model may not be representative of a broader TME context, we extended these findings to a pulmonary metastasis model by injecting MC38 spheroids intravenously. Without anti-CD40 treatment, the pulmonary disease outcome after MC38 spheroid injection was not different in WT and Keap1^flox/flox^ VavCre mice, suggesting that in the absence of immunomodulatory interventions, immunosurveillance does not significantly change tumor fate within the short observation period. However, while anti-CD40 reduced lung metastasis counts in WT and nearly abolished metastases in Nrf2^flox/flox^ VavCre mice, it failed to control disease in Keap1^flox/flox^ VavCre mice (figure 7F).

Because PD-1 is a clinically predominant immunotherapy target, we also administered anti‐PD-1 to MC38‐bearing WT and Nrf2^flox/flox^ LysMCre mice on day 8 after tumor implantation. After this treatment, the macrophage-specific Nrf2‐KO group displayed markedly smaller tumor volumes than WT. A mixed‐effects model on log‐transformed tumor volumes showed a significant genotype×day interaction (p=0.0006), consistent with considerably impaired tumor growth in Nrf2-KO animals (figure 7G).

Finally, we extrapolated our observations in the MC38 tumor cell model across three additional cancer cell lines. First, we found that subcutaneous tumors from TC-1 (lung adenocarcinoma) and B16 (melanoma) cancer cells were responsive to combined anti-CD40/anti-PD-1 immunotherapy in WT mice but resisted treatment in Keap1^flox/flox^ VavCre mice (figure 7H,I). Second, we used KP1.9 lung adenocarcinoma cells instead of MC38 cells in our pulmonary model. Again, anti-CD40 antibody treatment reduced the pulmonary disease burden in WT but not in Keap1^flox/flox^ VavCre mice (online supplemental figure 3).

Collectively, our data demonstrate that ablating Nrf2 in macrophages enhances the therapeutic effect of macrophage-directed (anti-CD40) and T-cell engaging (anti-PD-1) immunotherapies. In contrast, constitutively active NRF2 contributes to unchecked cancer progression.

## Discussion

Using a combination of high-definition spatial transcriptomics, scRNA-seq, and complementary genetic models, we found that in stressed tumors, TAMs display a spatially organized spectrum of phenotypes, extending from antigen-presenting pro-inflammatory cells at the tumor periphery to anti-inflammatory macrophages enriched in necrotic regions. This distribution is consistent with a gradient of ischemia, oxidative stress, and hemorrhage, which we confirmed to be conducive to NRF2 activation. Such a gradient of macrophage states illustrates a dynamic differentiation trajectory, with monocyte-derived macrophages adapting to regional stressors as they infiltrate deeper into the tumor. By revealing that NRF2-driven macrophages cluster near necrosis and suppress T-cell immunity, our study provides new mechanistic insights into the cellular architecture and functional complexity of the TME.

Our data align with and extend prior reports that macrophage functionality is context-dependent and influenced by local metabolic, hypoxic, and oxidative conditions. After administration of anti-CD40 antibodies, which enforces pro-inflammatory macrophage reprogramming,^18 29 30^ monocyte-derived macrophages expressing pro-inflammatory, antigen-presentation genes accumulate at the tumor margins. In contrast, anti-inflammatory Spp1^+^/Arg1^+^ macrophages, devoid of immunostimulatory functions, cluster near necrotic cores, underscoring the significance of the “functional tumor geography”. Interestingly, a comparable macrophage phenotype dichotomy emerges under irradiated conditions, implying a shared mechanism across different clinical interventions that induce necrosis or hemorrhage. These observations underscore how therapy-related tissue damage—whether from immunotherapies or high-dose radiation—can intensify local stress, ultimately giving rise to immunosuppressive macrophage subsets.

A key mechanistic insight is the partitioning of macrophages into pro-inflammatory Cxcl9^high^/MHC-II^high^ and anti-inflammatory Spp1^high^/Cxcl9^low^/MHC-II^low^ populations along an NRF2 activation gradient. In the necrotic tumor core, where cells face persistent oxidative and metabolic insults, NRF2 stabilizes and translocates to the nucleus, enabling macrophages to survive in this hostile environment. However, while this transcriptional program is cytoprotective, our data highlight that it restricts key macrophage immune functions. Cxcl9 is an IFN-induced macrophage gene with a strong T-cell attracting function,^31^ whereas Spp1 is a potent suppressor of cytotoxic T-cells through its interactions with CD44.^32^ Across various human tumors, we found a high NRF2-imprinted stress-TAM signature in Spp1+/Cxcl9− and a low NRF2-imprinted stress-TAM signature in Cxcl9+/Spp1− macrophages, suggesting that NRF2-driven macrophage polarization is a universal regulatory pathway. Recent studies linking a high Spp1:Cxcl9 index to poor clinical outcomes in multiple tumor types^19^ reinforce the notion that NRF2-governed TAMs are broadly detrimental.

Tumors grown in conditional Keap1 KO mice harbor macrophages skewed towards NRF2-imprinted stress-TAMs that are markedly less responsive to immune-stimulating signals. Instead of engaging in immune activation and productive cross-talk with T-cells, NRF2-imprinted stress-TAMs appear locked into an anti-inflammatory, immune-suppressive state. They downregulate MHC-II expression and key IFN-responsive genes, blunting their ability to prime and recruit T-cells.^33^ Simultaneously, NRF2-imprinted stress-TAMs accelerate tumor cell proliferation, facilitate the transition of cancer cells into an EMT state, and promote a TME, which impairs T-cell killing. This synergy of tumor progression and immune evasion further illustrates how therapy-induced necrotic zones create a protected habitat for malignant cells, reflecting a barrier to effective immunotherapy.^34^ Our findings reveal that the spatial organization of TAMs is not merely a descriptive characteristic of the TME but a determinant of systemic immune responses. Pro-inflammatory macrophages at the tumor periphery foster a microenvironment conducive to T-cell priming and recruitment, whereas the NRF2-imprinted immunosuppressive TAMs in necrotic cores correlate with reduced T-cell proliferation and function. This spatial-functional linkage underscores the importance of targeting localized stress responses to overcome systemic immunosuppression.

Critically, the tumor-promoting capacity of NRF2 extends beyond therapy-induced stress. In the spontaneous MMTV-PyMT breast cancer model, where endogenous T-cell activity usually restrains lesion outgrowth on the C57BL/6 background,^27^ tumors in conditional Keap1 KO hosts grew rapidly once palpable, whereas >60 % of WT tumors plateaued. Latency was unchanged, indicating that NRF2 does not affect tumor initiation but rather undermines immunosurveillance during progression.

A key translational relevance of our findings lies in their implications for immunotherapy. Macrophages occupy a central nexus in a bidirectional feedback loop with T-cells: pro-inflammatory macrophages potentiate T-cell activation through enhanced antigen presentation (MHC-II) and chemokine production (Cxcl9/Cxcl10). In contrast, activated T-cells secrete IFN-γ to reinforce macrophage polarization. Thus, modulating macrophage polarization can profoundly shape T-cell fate in patients with cancer—an area of immediate clinical interest.^35^ Paradoxically, treatment-related cell death, necrosis, or hemorrhage can amplify local stress, thereby activating NRF2 in macrophages.^5^ This negative feedback disrupts the beneficial macrophage–T-cell amplification loop: NRF2-imprinted stress-TAMs dampen antigen presentation, impair T-cell infiltration and function, and eventually subvert the intended immunostimulatory effect of therapy. Indeed, our experiments showed that enforcing an NRF2-imprinted stress state drastically diminished the therapeutic efficacy of anti-CD40-mediated macrophage reprogramming. In contrast, mice with macrophage-specific NRF2 deletion exhibited enhanced immunotherapeutic responses in anti-CD40 and anti-PD-1-based regimens. These observations suggest selectively targeting NRF2 or its downstream pathways in macrophages could overcome resistance and reinstate the positive T-cell macrophage crosstalk, which is crucial for robust tumor immunity.^36^ Consistent with our anti-CD40 findings, we also found that focused radiation therapy can intensify local tissue stress, again expanding Spp1^+^ macrophages in necrotic zones, highlighting a broader phenomenon relevant to multiple treatment modalities.

We recognize several limitations of our study. First, while our mouse models and spheroid co-cultures provide mechanistic insights, the full complexity of human tumors may involve additional signals, cellular interactions, and temporal dynamics not captured here. Further in vivo experiments and longitudinal studies will be necessary to understand the timing of macrophage phenotype shifts and their reversibility. It will also be essential to validate whether interventions to modulate NRF2 activity can safely and effectively enhance immunotherapeutic outcomes in more diverse tumor models and clinical settings. Second, we identified a clear link between necrosis and NRF2-imprinted phenotypes and defined hypoxia and the necrosis-associated toxin heme as potential drivers. Still, we have not exhaustively delineated which local factors most robustly engage the NRF2 axis and how other transcription factors, such as hypoxia-inducible factors (HIF-1 and HIF-2), may synergize with NRF2 to further enhance metabolic reprogramming and immunosuppression.^37^ Future studies will be required to unravel the relative contributions of necrotic versus non-necrotic stressors in vivo and to define the downstream signaling pathways and mechanisms by which NRF2-imprinted stress-TAMs rewire cancer cell growth, EMT state, and T-cell functions. Our study also lacks pharmacological validation, which will be critical for future clinical translation. Direct NRF2 inhibitors have only recently entered Phase I testing (eg, selective KEAP1 activators), whereas widely used tool compounds (eg, brusatol, ML385) have off-target or preclinical limitations. In this landscape, we deliberately prioritized clean, bidirectional genetic models, providing a mechanistic benchmark for expected direction and magnitude of benefit once selective agents suitable for immuno-oncology combinations mature.

Our findings establish an experimental framework linking therapy-induced stress—from immunotherapy or radiation—to immune evasion via NRF2-imprinted stress-TAMs. Our data reveal that the NRF2 activation state of macrophages forms a central regulatory node in a loop that can either promote tumor regression or, under NRF2-driven stress conditions, shield malignant cells from immune attack. By delineating how stress-induced macrophage states shape T-cell immunity and treatment outcomes, we provide a conceptual basis for strategies to neutralize the negative feedback imposed by NRF2-imprinted stress-TAMs. Looking ahead, inhibiting NRF2 emerges as a promising tactic to restore the macrophage–T-cell amplification cycle and enhance the efficacy of immunotherapies.

## Materials and methods

Detailed experimental protocols are provided in the online supplemental methods. A detailed Resource table is provided as an online supplemental table 1.

### Mouse strains

C57BL/6J: Charles River Laboratories. Ms4a3Cre: Dr Florent Ginhoux (SingHealth and Duke NUS, Singapore). Rosa26tdTomato mice: Jackson Laboratories. C57BL/6J-Spp1em1Msasn/J: Jackson Laboratories. Conditional Keap1 knockout mice: Keap1^tm2.Mym^^38^: RIKEN BRC. VavCre, LysMCre, Rag2^−/−^γc^−/−^, B6.Cg-Tg(TcraTcrb)425Cbn/J (OT-2), C57BL/6-Tg(TcraTcrb)1100Mjb/J (OT-1), B6.SJL-Ptprca Pepcb/BoyJ (CD45.1) mice: Swiss Immunological Mouse Repository (SwImMR). Conditional Nrf2 knockout mice: C57BL/6-Nfe2l2tm1.1Sred/SbisJ (Nrf2^flox^):^39 40^ Jackson Laboratories, crossed with VavCre or LysMCre mice. Control littermates without the Cre driver were used for experiments involving these mouse strains. MMTV-PyMT on C57Bl/6 background: Jackson Laboratories, bred with VavCre Keap1 flox/flox females. Conditional Keap1 KO and WT MMTV-PyMT females were used for tumor growth experiments. The Cd40flox/flox mouse strain was generated from the ES clone EPD0901_3_A02, obtained from the KOMP repository (www.komp.org), by the Wellcome Trust Sanger Institute as described previously.^41^ Cd40flox/flox mice were crossed with LysMCre mice.

### Statistics

Data plotting and statistical analysis were performed with Prism V.11 (GraphPad) and JMP 17 PRO (SAS). We used analysis of variance with Tukey-Kramer post-test to account for multiple comparisons and t-tests (two-tailed), as indicated. Tumor volumes measured repeatedly in individual mice were analyzed with linear mixed-effects models on the log scale to account for multiplicative growth. Models included time (day, continuous), group (treatment, genotype), and their interaction, with a random intercept for each mouse and a random slope for day when supported by the data (fitted by REML, statsmodels MixedLM in Python).

The primary outcome was the difference in growth rates between groups, estimated from the day×group interaction. Pairwise slope contrasts were tested with two-sided Wald t-tests and adjusted for multiple comparisons (Holm). Slopes were back-transformed to per cent change per day for interpretation.

We used the ARRIVE guideline to draft this manuscript;^42^ the ARRIVE reporting checklist is provided as a supplement.

## Supplementary material

10.1136/jitc-2025-013063 online supplemental file 1

10.1136/jitc-2025-013063 online supplemental file 2

10.1136/jitc-2025-013063 online supplemental file 3

10.1136/jitc-2025-013063 online supplemental file 4

## Data Availability

Data are available in a public, open access repository.
