# Supplementary material for: Stress-NRF2 response axis polarizes tumor macrophages and undermines immunotherapy
Source: J Immunother Cancer. 2025 Oct 31;13(10):e013063. doi: 10.1136/jitc-2025-013063 (PMC12581087; doi:10.1136/jitc-2025-013063)
Supplement: online supplemental file 1 [file jitc-13-10-s001.pdf]

Supplementary Methods

A stress-NRF2 response axis polarises tumor macrophages and undermines immunotherapy

Dominik J. Schaer[1], Nadja Schulthess-Lutz[1], Livio Baselgia[1], Kahrisan Kunasingam[1], Rok Humar[1], Kerstin Hansen[1], Melanie Eschment[1], Elena Duerst[1], Florence Vallelian*[1]

[1] Department of General Internal Medicine, University Hospital and University of Zurich, Zurich, Switzerland

*Correspondence:
Florence Vallelian MD
Department of Internal Medicine
University Hospital, Ramistrasse 100
CH-8091 Zurich, Switzerland
florence.vallelian@usz.ch

MATERIALS AND METHODS

Animals

C57BL/6J (JAX^{TM} strain) mice were obtained from Charles River Laboratories. Ms4a3Cre mice were obtained from Dr. Florent Ginhoux (SingHealth and Duke NUS, Singapore) and bred with *Rosa26tdTomato* mice (The Jackson Laboratory). C57BL/6J-Spp1em1Msasn/J mice were obtained from Jackson Laboratories. *Conditional Keap1 knockout mice: Keap1*$^{tm2.Mym}$ [1] mice were obtained from RIKEN BRC and crossed with VavCre or LysMCre mice, which were obtained from the Swiss Immunological Mouse repository (SwImMR). *Conditional Nrf2 knockout mice:* C57BL/6-*Nfe2l2tm1.1Sred/SbisJ* (*Nrf2*^{flox}) [2,3] mice were obtained from Jackson Laboratories and crossed with VavCre or LysMCre mice. Control littermates without the Cre driver were used for experiments involving these mouse strains. Transgenic MMTV-PyMT males in C57Bl/6 background were obtained from Jackson Laboratories and bred with VavCre Keap1 flx/flx females. Conditional Keap1 KO and WT MMTV-PyMT females were used for tumor growth experiments. *Rag2*$^{-/-}$*γc*$^{-/-}$ mice were obtained from the SwImMR. Other mice: B6.Cg-Tg(TcraTcrb)425Cbn/J (OT-2), C57BL/6-Tg(TcraTcrb)1100Mjb/J (OT-1), and B6.SJL-Ptprca Pepcb/BoyJ (CD45.1) were obtained from the SwImMR. CD45.1 OT-2 and CD45.1 OT-1 mice were obtained by crossing [4]CD45.1 mice with OT-2 and OT1 mice, respectively. The CD40fl/fl mouse strain was generated from the ES clone EPD0901_3_A02, obtained from the KOMP repository (www.komp.org), by the Wellcome Trust Sanger Institute (WTSI) as described previously.[4]. CD40 fl/fl mice were crossed with LysMCre mice.

All breeding colonies were housed and bred in the specific pathogen-free (SPF) animal facility at the Laboratory Animal Services Center (LASC) of the University of Zurich in individually ventilated cages. Animal experiments have been conducted at LASC. Male and female mice aged 7-12 weeks were used for all experiments, and all experiments with mice were performed according to animal experimentation licenses approved by the Swiss Federal Veterinary Office. For all studies, mice were randomly allocated to treatment groups, and the investigators were blinded to allocation during experiments and outcome assessment. The P.I. was aware of group allocation at the time of data analysis. All studies were conducted in accordance with the ARRIVE guidelines.[5]

Cell lines and primary cultures

Tumor cell line cultures

GFP-MC38 (donated by Gerhard Christofori, Department of Biomedicine, University of Basel, Basel, Switzerland) were cultured in RPMI-1640 medium supplemented with 10% fetal bovine serum (FBS, Gibco), 1% penicillin/streptomycin (P/S, Gibco), 1% nonessential amino acids (NEAA, Gibco) and 1% sodium pyruvate (Gibco). A cell line with homogeneous GFP expression was obtained by FACS sorting. Cell line authentication was performed before and after cell sorting by Short Tandem Repeat (STR) DNA genotype analysis (Microsynth, Balgach, Switzerland). GFP-OVA-MC38 (donated by Marianne Spalinger, Department of Gastroenterology, University of Zurich, Zurich, Switzerland) were cultured in DMEM medium (Gibco) supplemented with 10% fetal bovine serum (FBS, Gibco), 1% penicillin/streptomycin (P/S, Gibco), 1% nonessential amino acids (NEAA, Gibco) and 8.75ml 20% Glucose (Bichsel). THP-1 cells (InvivoGen) were cultured in RPMI 1640 medium supplemented with 10% FBS. KP1.9 cells (donated by Mikael Pittet, Ludwig Cancer Research, University of Geneva, Switzerland) and TC-1 cells (Cytion) were cultured in IMDM supplemented with 10% FBS, 1% P/S. B16 melanoma cells (ATCC) were cultured in DMEM with 10% FBS, P/S and L-glutamine. GFP-HT-29 cells (BioCat) were cultured in McCoy's 5A with 10% FBS. KP1.9 mEGFP cells were generated with Incucyte Nuclight Lentivirus reagent (Sartorius), followed by FACS sorting.

BM cultures

BM cells were isolated by flushing the femurs and tibias of 7- to 12-week-old mice and then passed through a 70-µm filter. The BM cells were plated at a density of 3 x 10^5 cells/ml on tissue culture-treated 60 mm UpCell dishes (Nunc™ UpCell™, ThermoFisher) in complete RPMI-1640 medium (10% fetal calf serum (FCS), 1% L-glutamine and 1% P/S) supplemented with 100 ng/ml recombinant mouse M-CSF (PeproTech). On day 3, half of the medium was replaced. Some cultures were treated on day 3 with 300 µM heme. Poly(I:C) (InvivoGen, 500 ng/ml) was added on day 6 for 24 hours. For experiments involving conditioned medium from tumor cells, BMDMs were seeded after washing at the end of the differentiation period in 12-well plates (TPP) in MC38 conditioned medium for 12 hours and lysed in RNA lysis buffer 1% β-mercaptoethanol for transcriptome analysis. For hypoxia experiments, BMDMs were cultured in high glucose complete RPMI-1640 medium under 0.2% O_2 concentrations from day 3 to day 7 using a workstation.

For the GM-CSF-supplemented cultures, fresh medium containing recombinant mouse GM-CSF (2×, 40 ng/ml)(PeproTech) was added on day 2. On day 3, half of the medium was removed, and new medium supplemented with GM-CSF (20 ng/ml) was added. The BM cells were harvested for analysis on day 5 from the temperature-responsive cell culture plates after cooling to room temperature. Cells were washed twice in PBS and centrifuged (300*g*, 10 min) before processing.

CRISPR-Cas9 knockout

We generated THP-1 KEAP1 knockout cells using CRISPR/Cas9 ribonucleoprotein (RNP) complexes. Three single guide RNAs (sgRNAs) targeting exon 2 of KEAP1 were used. RNPs were assembled by combining Cas9 (20 pmol/l, Synthego), with µL sgRNAs (30 pmol/l, Synthego) and electroporation enhancer (IDT) using the Amaxa 4D Nucleofector system (Lonza) with the SG Cell Line 4D Nucleofector Kit (Lonza). For each reaction, 3×10^5 cells were pelleted (400*g*, 5 min) and resuspended in 20 µl SG solution containing the RNP mixture. Electroporation was performed using the FF-100 program, and the cells were immediately transferred to 24-well plates in pre-warmed medium supplemented with the ROCK inhibitor Y27632 (Lucerna Chem). After 48 h recovery, viable single cells were isolated with a BD Aria III 4L (100 µm nozzle; BD Biosciences) and expanded in medium supplemented with 10% conditioned medium, 20% FBS, and ROCK inhibitor for one week. Editing efficiency and knockout validation were performed by PCR amplification of the targeted KEAP1 locus followed by Sanger sequencing.

THP-1 differentiation into macrophages

THP-1 cells were seeded in 6-well TTP plates and differentiated with PMA (100 ng/ml) for 48-72 hours. After PMA treatment, cells were washed with PBS to remove PMA, and then fresh medium was added. The cells were rested for 24 hours before stimulation with LPS (10 ng/ml, 6 hours) or spheroid formation.

Heme preparation for cell culture

Hemin (heme-chloride) was obtained from Frontier Scientific (Newark). Batches were tested endotoxin-free and prepared for cell treatments as described.

3D tumor spheroid production, culture, and analysis

Single-spheroid culture

5 × 10^3 GFP-MC38 or TC-1 cells ± BMDMs (at a 1:1 ratio), 1.5 × 10^3 GFP-HT29 ± THP-1 cells (at a 1:1 ratio) were seeded in 100 µl tumor cell culture medium in 96-well Ultralow Attachment Plate PrimeSurface® 3D Culture Spheroid plates (S-BIO).

Multispheroid culture in microwell plates

GFP-MC38 cells (5 x 10^4) ± BMDMs (at a 1:1 ratio) were seeded in 800 µl of tumor cell medium with M-CSF (100 ng/ml) in a 24-well SphericalPlate® 5D microwell (Axonlab). On day 3, 800 µl of fresh culture cell medium was added.

Quantification of spheroid growth and invasion

Single spheroids were imaged in the cell culture incubator with an IncuCyte S3 instrument (Sartorius). Green fluorescence and phase contrast images of the spheroids were acquired every 3-4 hours for seven to ten days. The area and fluorescence intensities of the images were measured using the IncuCyte Spheroid Software Module (Sartorius). Data are reported as spheroid fluorescence intensity integrated across the spheroid area (for tumor cells expressing a fluorescent protein) or as spheroid area.

T cell assays

CD4+ T and CD8+ T cell isolation and CFSE labeling

Lymphocyte T cells were positively enriched from spleen single-cell suspensions using CD4 or CD8 enrichment kit (Thermofisher) according to the manufacturer's instructions. Isolated CD4+ T or CD8+ T cells were labeled with CFSE (Thermo Fisher) at RT for 20 min, washed with RPMI Medium and counted before use. The final purity, confirmed by flow cytometry, was >95%.

OT-2 assay

A total of 2x10^4 FACS-sorted CD45+ F4/80+ TAMs or GM-CSF stimulated BM cells were plated in 96-well round-bottom plates (Falcon), pulsed or not with 1 µg/ml $Ova^{323–339}$ peptides (Sigma) for 45 min at 37 °C, and washed three times with PBS. Subsequently, 1 × 10^5 CFSE-labeled naive CD4+ T cells isolated from spleens of OT-2 mice were added to the BM cells in complete RPMI-1640 medium and cocultured at 37 °C. CFSE dilution was assessed by flow cytometry after three days.

Adoptive Transfer of OT-1 CD8+ T cells

2×10^6 CFSE-labeled CD8+ T cells isolated from OT-1 × CD45.1 mouse spleens were injected intravenously via the tail vein into conditional Keap1 KO mice and WT littermates. The mice were challenged intravenously with agonistic anti-CD40 antibody (20 mg/kg, InVivoPlus, clone FGK4.5). The mice were sacrificed three days later, and CD45.1+ CD8+ T cells were analyzed by flow cytometry.

CD8+ T cell activation

1×10^6 CFSE-labeled CD8+ T cells isolated from OT-1 mouse spleens were activated with anti-CD3/anti-CD28 Dynabeads (Invitrogen) and incubated overnight in complete RPMI-1640 medium or in supernatant of mixed cell spheroids that have been cultured in microwell plates for 5 days.

T-cell killing assay

5×10^3 GFP-OVA-MC38 cells were seeded into 96-well flat bottom plates (TTP) and cultured for 20 hours. Subsequently, 5×10^3 activated OVA-specific CD8+ T cells were added to the tumor cells, and green fluorescence and phase-contrast images were acquired every 2 hours. The area and fluorescence intensities of the images were measured using the IncuCyte Cell-by-Cell Software Module (Sartorius). Data are reported as fluorescence intensity integrated across the area.

Tissue/cell preparation and digestion

Tumor digestion

Subcutaneous tumors were excised and minced using a scalpel on a sterile Petri dish. The minced tumor tissue was then dissociated in 3 ml of digestion medium (RPMI medium (Gibco) supplemented with 25 µg/ml Liberase™ (Roche) and 40 µg/ml DNase I (Roche; 2000 U/ml). This mixture was incubated for 30-45 minutes in a water bath at 37°C. The dissociated tumor cells were filtered through a 70-µm cell sieve and washed with PBS containing 2 mM EDTA to halt the digestion process. Following the lysis of red blood cells using RBC lysis buffer (BioLegend), the cells were washed again and immediately utilized.

Macrophage isolation from digested tumors

Anti-rat IgG Dynabeads (Invitrogen) were washed and incubated with rat anti-mouse F4/80 IgG2a antibodies (BD Biosciences) and CD11b IgG2b antibodies (BioLegend) at a ratio of 3.33 µg of antibody per 50 µl of Dynabeads. Single-cell suspensions from tumors were incubated with anti-F4/80-coated (for RT-qPCR or RNA bulk sequencing experiments) or anti-CD45 coated Dynabeads (for scRNA-seq experiment) on a rotating wheel at 4°C for 30 min. After incubation, a positive selection of Dynabead-bound single-cell suspensions was performed on a DynaMag magnet (Invitrogen) with three washing steps, as the manufacturer's instructions suggested.

Spheroid digestion

Spheroids were dissociated in 2 ml digestion medium (RPMI medium (Gibco) + 25 µg/ml Liberase™ (Roche) + 40 µg/ml DNase I (Roche; 2000 U/ml) and incubated for 30-45 min in a water bath at 37°C with gentle shaking every 5 min. Then, 4 ml PBS + 0.04% BSA was added to stop the digestion. Digested spheroids were used immediately.

Mouse models

Lung metastasis model in mice

Approximately 750 spheroids were collected from microwell plates (equal to the content of one macro well) 4 days post-spheroid formation and injected intravenously into the tail vein of recipient mice. Agonistic anti-CD40 treatment (20 mg/kg, InVivoPlus, clone FGK4.5) or an isotype control antibody was administered intravenously according to the treatment protocol. Two or three weeks post-injection, the lungs of anesthetized mice were perfused with PBS through the right ventricle and the trachea and collected for whole-organ fluorescence imaging with a Zeiss Discovery V8 stereomicroscope and histology.

KP1.9 lung tumors

KP1.9 lung tumors were induced by intravenous injection of KP1.9 spheroids collected from microwell plates (equal to the content of one macro well) one day post-spheroid formation into the tail vein of recipient males. Agonistic anti-CD40 treatment (20 mg/kg, InVivoPlus, clone FGK4.5) or an isotype control antibody was administered intravenously according to the treatment protocol (day 7-10-13). Three weeks post-injection (day 21), the lungs of anesthetized mice were perfused with PBS through the right ventricle and the trachea and collected for histology.

Subcutaneous tumor growth models in mice

Once confluent, MC38, TC-1, or B16-F10 tumor cells were harvested using 5 mM EDTA (Gibco) (4 min at 37°C). MC38 (2×10^6) , TC-1 cells (1×10^6) or B16 cells (1×10^6) in culture medium were mixed with Geltrex (Thermo Fisher) and injected subcutaneously into the mouse flanks. Agonistic anti-CD40 treatment (20 mg/kg, Bio X Cell, clone FGK4.5), antagonistic anti-PD-1 (CD279) (10 mg/kg, Bio X Cell) or an isotype control antibody was administered intravenously according to treatment protocol. Mice were euthanized, and tumors were collected at defined time points after antibody administration. Tumors were then digested or fixed in 10% formalin and stored at room temperature. Tumors were allowed to grow until they reached 1000 mm^3 or until skin necrosis occurred.

Mammary tumor growth was measured by digital 3D topography in WT and conditional Keap1 KO MMTV-PyMT females. Mammary tumors were allowed to grow until they reached 1000 mm^3 or until skin necrosis occurred.

Tumor volume measurements

All tumor volumes were measured non-invasively by an investigator blinded to treatment or genotype with a Peira TM900 device, which extracts volumes from high resolution stereo-3D images.

Flow cytometry

Cells were preincubated with Mouse BD Fc Block™ (≤ 1 µg/million cells in 100 µl, BD Biosciences) at 4°C for 10 min. The following antibodies were purchased from BD Biosciences: anti-CD45 (clone 30-F11), anti-F4/80 (clone T45-2342), and anti-I-A/I-E (clone M5/114.15.2). The following antibodies were purchased from BioLegend: anti-CD45.1 (clone A20), anti-CD45.2 (clone 104), anti-CD4 (clone GK1.5), anti-CD8 (clone 53-6.7), and anti-CD69 (clone H1.2F3). Corresponding isotype-matched irrelevant specificity controls were purchased from BD, and BioLegend. Multiparameter analysis was performed with an LSRFortessa analyzer (BD Biosciences). The data were analyzed using FlowJo software (version 10.7.1). Cell sorting was performed on a BD FACSAria III 4L.

Histology

Organ fixation for paraffin embedding and microtome sectioning

Mice were anesthetized by intraperitoneal injection of ketamine (80 mg/kg), xylazine (16 mg/kg), and acepromazine (3 mg/kg) and transcardially perfused with cold PBS. Organs were placed in 10% formalin and transferred to 70% ethanol after 24 hours before embedding in paraffin blocks. Microtome sections (2-2.5 µm) of each organ were cut for H&E staining and immunohistochemistry.

Immunohistochemistry

GFP staining: Tissue sections were incubated overnight with a goat anti-GFP antibody (Abcam) diluted 1:1000, followed by a biotinylated horse anti-goat secondary antibody (Vector) diluted 1:500. *Anti-F4/80 staining*: Tumor sections were incubated overnight with a rat anti-mouse F4/80 antibody (Bio-Rad, MCA497G) diluted 1:80, followed by a biotinylated goat anti-rat secondary antibody (Vector, BA9401) diluted 1:500.

All immunohistochemical sections were rinsed in 0.1 M phosphate buffer, pH 7.4, and incubated with diaminobenzidine (DAB, Abcam) for 2-5 min. After incubation, sections were washed in deionized water and lightly counterstained with hematoxylin solution, according to Mayer (Sigma).

Microscopy image acquisition and analysis

A Zeiss Axio Scan Z1 Slidescanner microscope imaged whole-lung sections and subcutaneous tumors. Images were analyzed using Qupath and ImageJ. Brightness, contrast, and color tone (for single-channel fluorescence images) were adjusted with Adobe Lightroom software version 8.1. using identical settings for all images of an experiment.

Sequencing-based workflows and data analysis

Bulk RNA sequencing

RNA was extracted from macrophages using the RNeasy Mini kit (Qiagen) according to the manufacturer's protocol, including on-column DNase I treatment. RNA quality was validated on an Agilent Technologies 4150 Tapestation using RNA Screentapes, and only samples with an RNA integrity number (RIN) of > 9 were used for sequencing. cDNA libraries were generated at the Functional Genomics Center Zurich (FGCZ) from RNA samples using the Illumina Stranded mRNA Prep ligation kit following the manufacturer’s instructions. The quality and concentration

of the libraries were determined using an Agilent Technologies 4200 Tapestation with DNA Screentapes. The libraries were pooled in equimolar amounts and sequenced on an Illumina NovaSeq X Plus sequencer (paired-end 150 bp) with a depth of at least 20 million reads per sample.

Bulk RNA sequencing data analysis

Reads were aligned to the reference genome Ensembl GRCm38.p5 Release 91 using STAR (v2.7.0e).[6] The quality of alignment was evaluated using Samtools (v1.9). [7] Counts were obtained using the featureCounts function of the Rsubread package (v1.22.2).[8] Differential expression analysis was performed with the DESeq2 R package (v1.26.0)[9].

scRNA-seq sample preparation

Single-cell suspensions were fixed following the demonstrated protocol Fixation of cells & Nuclei for Chromium Fixed RNA Profiling (10X Genomics, CG000478) and processed for long-term storage at -80°C. After storage, up to 2 Mio cells per sample were hybridized with unique single Mouse WTA probes using BC001-004 according to the demonstrated protocol Chromium Fixed RNA Profiling Reagent Kits for Multiplexed Samples (10X Genomics, CG000527). Following gene expression library construction, ready-made libraries were sequenced at the Functional Genomics Center Zurich (FGCZ) on an Illumina NovaSeq X Plus system following the recommendations of 10X Genomics.

scRNA-seq sample analysis

Downstream analysis was performed in Python (version 3.10.10) with Scanpy (1.9.2).

<u>Read alignment</u>

Reads were aligned to the mouse reference genome GENCODE GRCm39 (Release_M31-2023-01-30) using CellRanger (version 7.2.0).

<u>Quality Control and Preprocessing</u>

To assess the quality of the cells, the following covariates were considered: number of genes expressed in a cell (*n_genes_by_counts*), number of counts per cell (*total_counts*), and percentage of mitochondrial RNA (*pct_counts_mt*). Cells that expressed fewer than *min_genes*

or more than *max_genes* were filtered out. Cells with a percentage of mitochondrial RNA greater than *max_pct_mt* were considered dead and removed from the analysis. Genes that were expressed by fewer than *min_cells* cells were excluded. See below for the cutoff values used in each experiment. The count data were normalized so that every cell has the same total count after normalization (sc.pp.normalize_total) and log(x+1) (`sc.pp.log1p`) transformed, yielding normalized expression values. For Fig.5 an algorithm based on deconvolving size factors from cell pools implemented in the R package scran (`calculateSumFactors`)[10] was used due to the higher complexity of the experiment data.

<u>Data integration</u>

Multiplexed samples were merged into one dataset by simple concatenation. Additionally, samples from different experiments were integrated using the harmony (`sc.external.pp.harmony_integrate`) [11] or the scanorama algorithm [12], depending on the experimental setup and its complexity.

<u>Dimensionality reduction and clustering</u>

For dimension reduction, the following steps were performed using the Python package Scanpy: identifying highly variable genes (`sc.pp.highly_variable_genes`), performing PCA using highly variable genes (`sc.tl.pca`), computing the neighborhood graph (`sc.pp.neighbors`) and computing the UMAP (`sc.tl.umap`). The cells were clustered using Leiden clustering (`sc.tl.leiden`), which depends on the neighborhood graph. The resolution of the Leiden clustering was chosen so that a biologically meaningful number of clusters was produced.

<u>Cell type annotation and functional classification</u>

To identify cell types, we analyzed the expression of marker genes and other differentially expressed genes (`sc.tl.rank_genes_groups` with `method = 'wilcoxon'`). GSEA was performed to assess functional and biological process-related differences between clusters or conditions. First, genes were ranked using the output of the Wilcoxon rank-sum test (`rank = -log10(adj. p value)*sign(logfoldchange)`) and then fed to the GSEA algorithm implemented in the Python package decoupler [13]; [14] (`decoupler.get_gsea_df`), resulting in a normalized enrichment score (NES) and a false discovery rate (FDR) per gene set.

For transcription factor analysis with the TRRUST_Transcription_Factors_2019 gene set database and for scoring enrichment of gene sets per cell , the AUCell algorithm (`decoupler.run_aucell`) was used.

Visium HD CytAssist Spatial Gene Expression for FFPE

Tissues were processed as described in the section titled organ fixation for paraffin embedding and microtome sectioning. Microtome sections on regular glass slides were deparaffinized and stained for H&E as described in Visium HD FFPE Tissue Preparation Handbook (10X Genomics, CG000684). Regions of interest on the tissue sections were chosen using H&E staining and aligned in the tissue slide cassette (6.5 mm). After destaining and decrosslinking, sections were immediately subjected to probe hybridization overnight followed by probe ligation, release and extension and spatial library construction as described in the user guide Visium HD Spatial Gene Expression Reagents Kits (10X Genomics, CG000685). Ready-made libraries were sequenced at the Functional Genomics Center Zurich (FGCZ) on an Illumina NovaSeq X Plus system using paired-end 150 bp read configuration.

Spatial transcriptomics analysis

Downstream analysis was performed in Python (version 3.10.10) with Scanpy (1.9.2) [15]. Each sample was processed individually.

<u>Read alignment</u>

Reads were aligned to the mouse reference genome Ensembl GRCm39 (Release_106-2022-07-05) using SpaceRanger (version 2.1.0).

<u>Nuclei Segmentation and Custom Binning of Visium HD Gene Expression Data</u>

We reassigned the barcodes to cell nuclei to extract the macrophages from the spot-based Visium HD data to create single-cell-like data. To achieve this, the cell nuclei were segmented based on a microscopy image of the H&E stained tissue as described in [https://www.10xgenomics.com/analysis-guides/segmentation-visium-hd]. After normalization (*min_percentile*, *max_percentile*) of the H&E image, a pre-trained model (StarDist2D: 2D_versatile_he) predicts the cell nuclei. Each barcode is then assigned to a cell nucleus, and the counts are aggregated. This results in a single-nucleus resolution of the data.

Quality Control, Preprocessing, Dimensionality Reduction

Cell nuclei that contain less than *min_total_counts* or their area is bigger than *max_area* are removed from the dataset. The data was normalized (sc.pp.normalize_total) and log(x+1)-transformed (sc.pp.log1p) to generate gene expression data.

Macrophages were annotated by scoring (sc.tl.score_genes) the gene expression of tissue-specific *macrophage marker genes*.

For further analysis, only cells labeled as macrophages were considered.

Based on the top 2000 highly variable genes (sc.pp.highly_variable_genes), a PCA was performed (sc.tl.pca), and a neighborhood graph was constructed (sc.tl.neighbors).

TAM-NRF2 scoring

Using the gene sets obtained from the differential gene expression analysis of bulk RNA-seq of F4/80$^+$ TAMs between Keap1$^{flox/flox}$ VavCre and WT mice, a combined TAM-Nrf2-on/TAM-Nrf2-off score was calculated (sc.tl.score_genes).

Statistics

Data plotting and statistical analysis were performed with Prism 11 (GraphPad) and JMP 17 PRO (SAS). We used ANOVA with Tukey–Kramer posttest to account for multiple comparisons and t-tests (two-tailed), as indicated. Tumor volumes measured repeatedly in individual mice were analyzed with linear mixed-effects models on the natural-log scale to account for multiplicative growth. Models included time (day, continuous), group (treatment, genotype), and their interaction, with a random intercept for each mouse and a random slope for day when supported by the data (fitted by REML, statsmodels MixedLM in Python).

REFERENCES

1. Taguchi K, Maher JM, Suzuki T, Kawatani Y, Motohashi H, Yamamoto M. Genetic analysis of cytoprotective functions supported by graded expression of Keap1. Mol Cell Biol. 2010;30:3016–26.

2. Kong X, Thimmulappa R, Craciun F, Harvey C, Singh A, Kombairaju P, et al. Enhancing Nrf2 pathway by disruption of Keap1 in myeloid leukocytes protects against sepsis. Am J Respir Crit Care Med. 2011;184:928–38.

3. Reddy NM, Potteti HR, Mariani TJ, Biswal S, Reddy SP. Conditional deletion of Nrf2 in airway epithelium exacerbates acute lung injury and impairs the resolution of inflammation. Am J Respir Cell Mol Biol. 2011;45:1161–8.

4. Pfefferlé M, Ingoglia G, Schaer CA, Hansen K, Schulthess N, Humar R, et al. Acute Hemolysis and Heme Suppress Anti-CD40 Antibody-Induced Necro-Inflammatory Liver Disease. Front Immunol. 2021;12:680855.

5. Percie du Sert N, Hurst V, Ahluwalia A, Alam S, Avey MT, Baker M, et al. The ARRIVE guidelines 2.0: Updated guidelines for reporting animal research. PLoS Biol. 2020;18:e3000410.

6. Dobin A, Davis CA, Schlesinger F, Drenkow J, Zaleski C, Jha S, et al. STAR: ultrafast universal RNA-seq aligner. Bioinformatics. 2013;29:15–21.

7. Danecek P, Bonfield JK, Liddle J, Marshall J, Ohan V, Pollard MO, et al. Twelve years of SAMtools and BCFtools. Gigascience. 2021;10:giab008.

8. Liao Y, Smyth GK, Shi W. The R package Rsubread is easier, faster, cheaper and better for alignment and quantification of RNA sequencing reads. Nucleic Acids Res. 2019;47:e47.

9. Love MI, Huber W, Anders S. Moderated estimation of fold change and dispersion for RNA-seq data with DESeq2. Genome Biol. 2014;15:550.

10. Lun ATL, McCarthy DJ, Marioni JC. A step-by-step workflow for low-level analysis of single-cell RNA-seq data with Bioconductor. F1000Res. 2016;5:2122.

11. Korsunsky I, Millard N, Fan J, Slowikowski K, Zhang F, Wei K, et al. Fast, sensitive and accurate integration of single-cell data with Harmony. Nat Methods. 2019;16:1289–96.

12. Hie B, Bryson B, Berger B. Efficient integration of heterogeneous single-cell transcriptomes using Scanorama. Nat Biotechnol. 2019;37:685–91.

13. Badia-I-Mompel P, Vélez Santiago J, Braunger J, Geiss C, Dimitrov D, Müller-Dott S, et al. decoupleR: ensemble of computational methods to infer biological activities from omics data. Bioinform Adv. 2022;2:vbac016.

14. Fang Z, Liu X, Peltz G. GSEApy: a comprehensive package for performing gene set enrichment analysis in Python. Bioinformatics. 2023;39:btac757.

15. Wolf FA, Angerer P, Theis FJ. SCANPY: large-scale single-cell gene expression data analysis. Genome Biol. 2018;19:15.
