# Supplementary material for: Stress-NRF2 response axis polarizes tumor macrophages and undermines immunotherapy
Source: J Immunother Cancer. 2025 Oct 31;13(10):e013063. doi: 10.1136/jitc-2025-013063 (PMC12581087; doi:10.1136/jitc-2025-013063)
Supplement: online supplemental file 2 [file jitc-13-10-s002.pdf]

Supplementary Figures

A stress-NRF2 response axis polarises tumor macrophages and undermines immunotherapy

Dominik J. Schaer[1], Nadja Schulthess-Lutz[1], Livio Baselgia[1], Kahrisan Kunasingam[1], Rok Humar[1], Kerstin Hansen[1], Melanie Eschment[1], Elena Duerst[1], Florence Vallelian*[1]

[1] Department of General Internal Medicine, University Hospital and University of Zurich, Zurich, Switzerland

*Correspondence:
Florence Vallelian MD
Department of Internal Medicine
University Hospital, Ramistrasse 100
CH-8091 Zurich, Switzerland
florence.vallelian@usz.ch

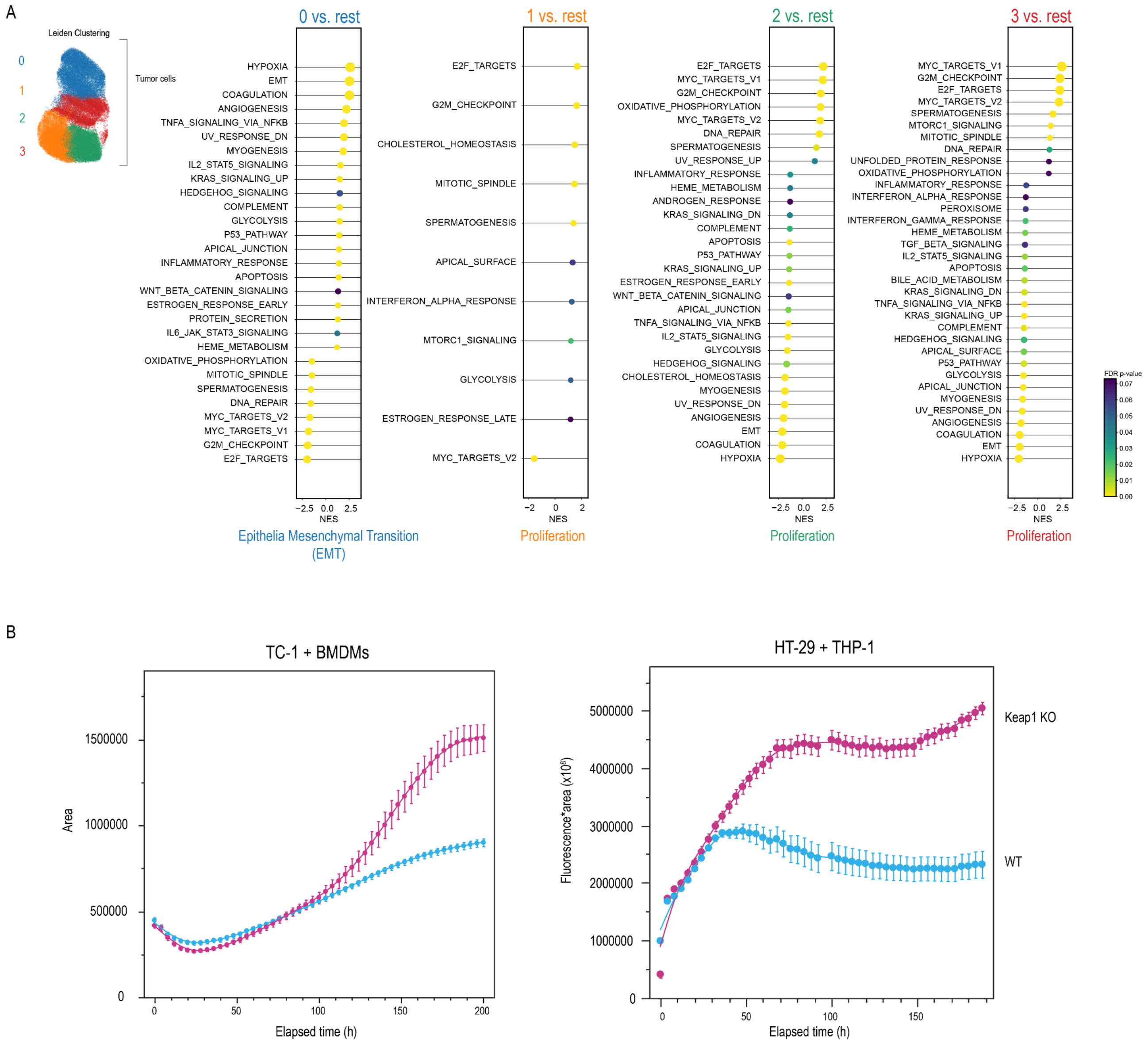

Supplementary Figure 1

A. Multiplexed scRNA-seq at 24 h and 120 h post-spheroid formation (Figure 6E). Leiden clustering of the integrated data and GSEA of the differential expressed genes.

B. Spheroid co-culture model: WT or Keap1 KO BMDMs mixed with TC-1 cells (left), and WT or Keap1 KO THP1 mixed with GFP-HT29 cells (right) were cultured in ultra–low-attachment plates. Live-cell microscopy tracks spheroid size (area or area × GFP intensity). Spheroids with Keap1 KO macrophages grow faster than those with WT macrophages. Data as mean ± SE of 8-10 replicates, each analyzed within on experiment

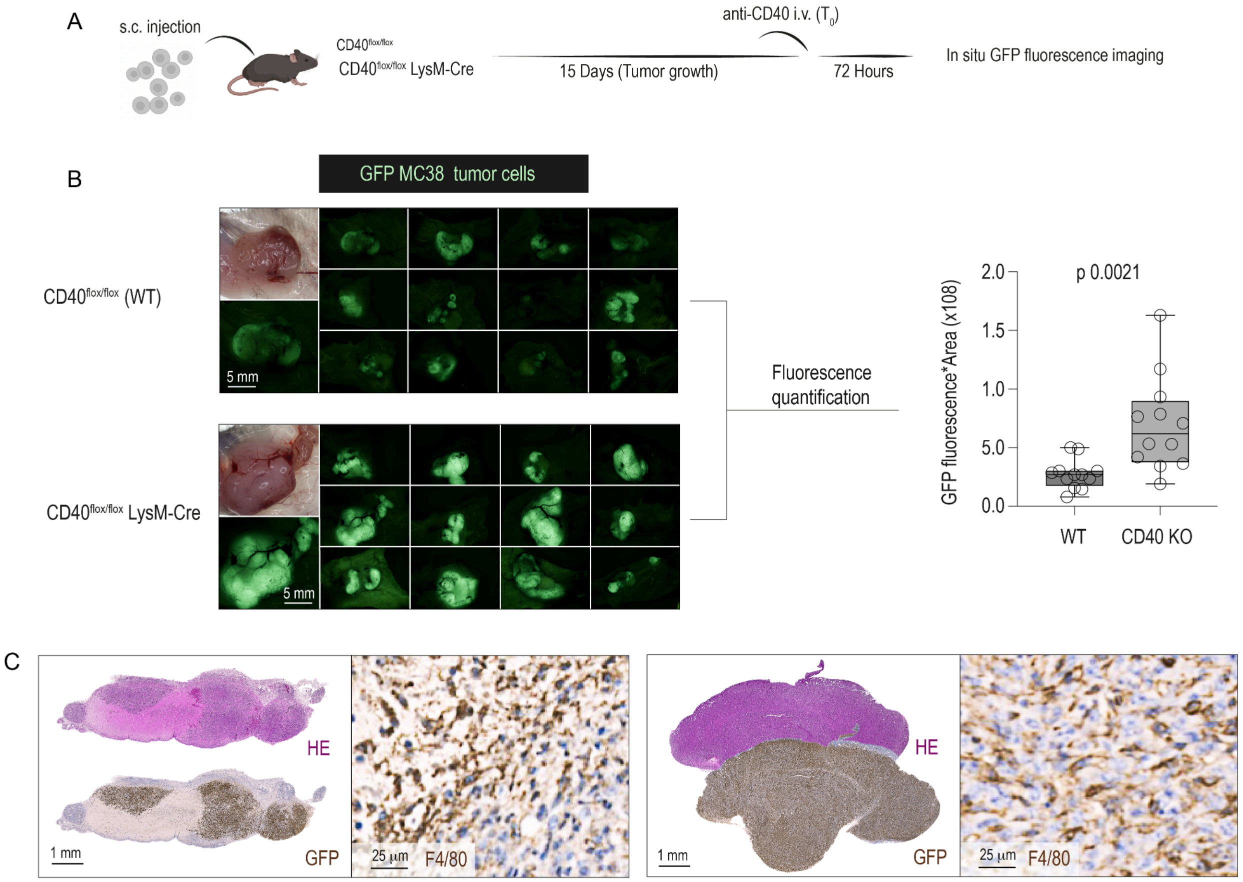

Supplementary Figure 2

A. MC38 tumors were grown s.c. in CD40$^{flox/flox}$ LysM versus WT mice. On day 15, mice received anti-CD40 treatment administered i.v. Three days after the second dose, tumors were collected for in situ GFP fluorescence imaging and histology.

B. Left and right**:** Bright-field and GFP fluorescence images visualizing MC38 tumors in situ. Middle: GFP fluorescence intensity integrated across the tumor area. Each dot represents one tumor grown on the right and left flank of a mouse (n = 12 tumors; mean ± SD, t-test).
C. Representative H&E-, anti-GFP and anti-F4/80 stained-MC38 tumor sections.

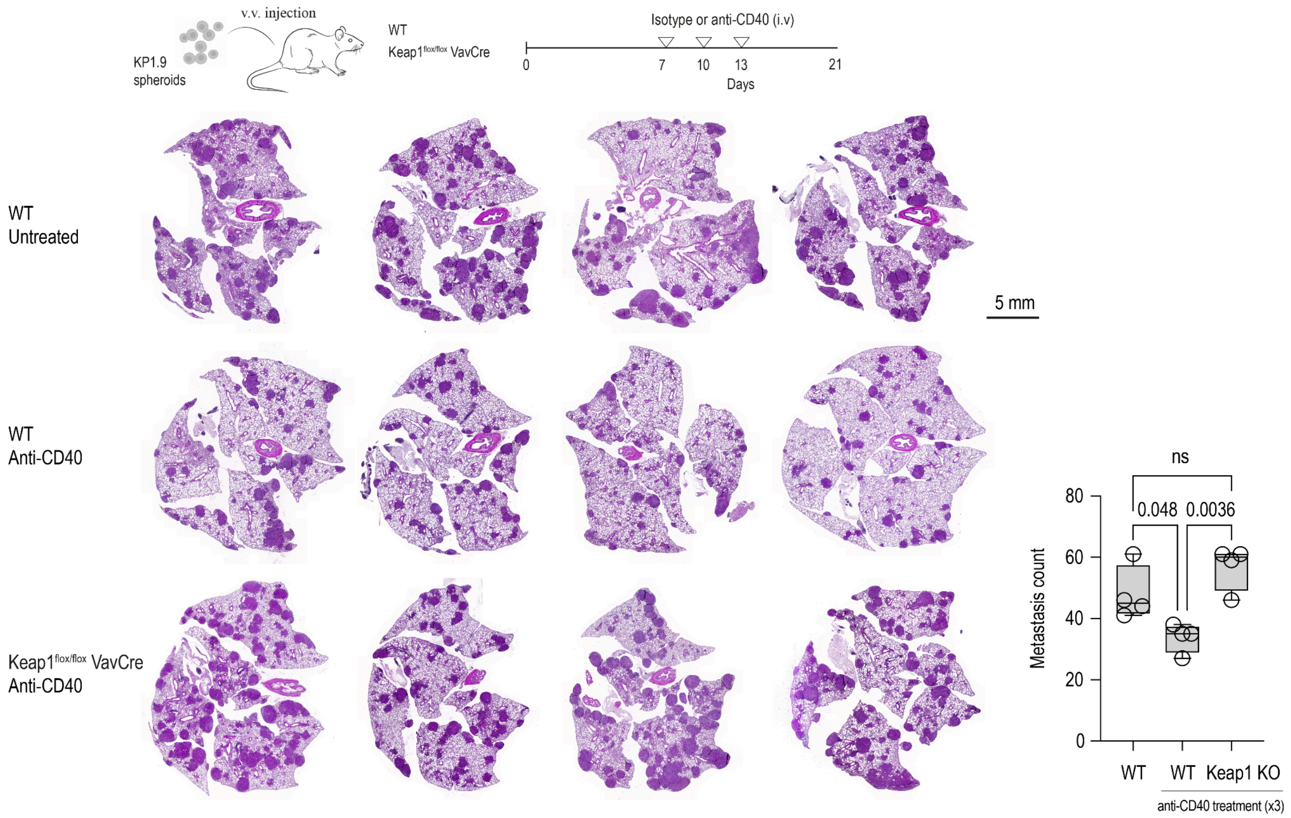

Supplementary Figure 3

A. KP1.9 lung tumors were grown in Keap1$^{flox/flox}$ VavCre versus WT mice. On day 7, 10, and 13 mice were treated by i.v. anti-CD40 antibody. On day 21, lungs were analyzed by histology (H&E staining) and metastases were manually counted by a blinded investigator. Anti-CD40 therapy fails to control tumor spread in conditional Keap1 KO mice. Each dot represents one mouse (n=4 pro condition, mean ± SD, ANOVA with Tukey–Kramer post-test).
