# Supplementary material for: Stress-NRF2 response axis polarizes tumor macrophages and undermines immunotherapy
Source: J Immunother Cancer. 2025 Oct 31;13(10):e013063. doi: 10.1136/jitc-2025-013063 (PMC12581087; doi:10.1136/jitc-2025-013063)
Supplement: online supplemental file 3 [file jitc-13-10-s003.pdf]

Schaer et al. A stress-NRF2 response axis polarizes tumor macrophages and undermines immunotherapy - Resource TABLE

Mouse strain	Company
C57BL/6J	Charles River
Ms4a3Cre	Prof. Florent Ginhoux (SingHealth and Duke NUS, Singapore)
LysMCre	Swiss Immunological Mouse repository (SwImMR)
VavCre	Swiss Immunological Mouse repository (SwImMR)
Rag2−/−γc−/−	Swiss Immunological Mouse repository (SwImMR)
B6.Cg-Tg(TcraTcrb)425Cbn/J (OT-2)	Swiss Immunological Mouse repository (SwImMR)
C57BL/6-Tg(TcraTcrb)1100Mjb/J (OT-1)	Swiss Immunological Mouse repository (SwImMR)
B6.SJL-Ptprca Pepcb/BoyJ (CD45.1)	Swiss Immunological Mouse repository (SwImMR)
C57BL/6J-Spp1em1Msasn/J	Jackson Laboratory Strain #:033731
CD40fl/fl	Generated in our laboratory
Rosa26tdTomato mice	Jackson Laboratory Strain #:007914
Keap1tm2.Mym	RIKEN BRC
C57BL/6-Nfe2l2tm1.1Sred/SbisJ (Nrf2flox)	Jackson Laboratory Strain #:025433

Tumor lines	Company
GFP+ MC38 colon adenocarcinoma cells	Donated by Gerhard Christofori, Department of Biomedicine, University of Basel
GFP-OVA-MC38	Donated by Marianne Spalinger, Department of Gastroenterology, University of Zurich
THP-1-Blue™ KI-IP10	InvivoGen, #thpb-ip10kilc
KP1.9 human monocytic leukemia	Group Mikael Pittet University of Geneva
TC1 murine Lungepithelia	Cytion (305388)
HT-29 human colorectal adenocarcinoma	BioCat (SL006-GVO-GC)
B16 F10 murine melanoma	ATCC (CRL-6475)

Reagents for in vivo or in vitro treatments

Product name	Company	Product number
InVivoPlus anti-mouse CD40	Bio X Cell	BP0016-2
InVivoMab anti mouse PD-1 (CD279)	Bio X Cell	BE0146
InVivoPlus rat IgG2a isotype control	Bio X Cell	BP0089
InVivoMAb rat IgG2a isotype control	Bio X Cell	BE0089
Ketamine	Graeub	QN01AX03
Xylazine	Bayer	QN05CM92
Acepromazine	Fatro	QN05AA04

Antibodies for flow cytometry

Targeted antigen	Clone	Fluorochrome	Species	Company	Product number
F4/80	T45-2342	Allophycocyanin (APC)	Rat	BD	566787
CD45	30-F11	PE-Cy7	Rat	BD	552848
CD45.1	A20	Brilliant violet 711	Mouse	Biolegend	110739
CD45.2	104	Pacific blue	Mouse	Biolegend	109820
CD4	GK1.5	eFluor 450	Rat	ThermoFisher	48-0041-82
CD4	GK1.5	Phycoerythrin (PE)	Rat	Biolegend	100408
CD8a	53-6.7	Phycoerythrin (PE)	Rat	Biolegend	100708
I-A/I-E	M5/114.15.2	Brilliant violet 421	Rat	BD	562564
CD69	H1.2F3	FITC	Rat	BioLegend	104506
APC Rat IgG2a κ Isotype Control	R35-95	Allophycocyanin (APC)	Rat	BD	553932
PE-Cy™7 Rat IgG2b, κ Isotype Control	A95-1	PE-Cy7	Rat	BD	552849
Mouse IgG2a, κ Isotype Ctrl Antibody	MOPC-173	Brilliant violet 711	Rat	Biolegend	400272
Mouse IgG2a, κ Isotype Ctrl Antibody	MOPC-173	Pacific blue	Rat	Biolegend	400235
Rat IgG2b, λ Isotype Ctrl Antibody	G013B8	Phycoerythrin (PE)	Rat	Biolegend	403804
Rat IgG2a, λ Isotype Ctrl Antibody	G013C12	Phycoerythrin (PE)	Rat	Biolegend	402304
BV421 Rat IgG2b, κ Isotype Control	R35-38	Brilliant violet 421	Rat	BD	562603
Armenian Hamster IgG Isotype Ctrl Antibody	HTK888	FITC	Rat	BioLegend	400906
Rat IgG2b kappa Isotype Control	eB149/10H5	eFluor 450	Rat	ThermoFisher	48-4031-82

Primary antibodies for immunofluorescence or IHC

Targeted antigen	Clone	Fluorochrome	Species	Company	Product number
F4/80	CI:A3-1	-	Rat	Biorad	MCA497G
Anti-GFP antibody	Polyclonal	-	Goat	abcam	ab5450

Secondary antibodies for immunofluorescence or IHC

Targeted antigen	Clone	Fluorochrome	Species	Company	Product number
Anti-Goat IgG (H+L)	-	Biotin	Horse	Vector Laborat	BA-95001.5
Anti-Rat IgG	-	Biotin	Goat	Vector Laborat	BA-9401-.5

Fluorescent dyes

Product name	Company	Product number
CellTrace™ Far Red Cell Proliferation Kit, for flow cytometry	Thermofisher	C34564
Incucyte® Nuclight Green Lentivirus 2.0 (puro)	Sartorius	BA-04888

Magnetic beads, magnets and associated antibodies

Product name	Company	Product number
Dynabeads™ Sheep anti-Rat IgG	Invitrogen	11035
Purified Rat anti-mouse F4/80 IgG2a antibodies (clone T45-2342)	BD biosciences	565409
Purified anti-mouse CD45 Antibody	BioLegend	103102
DynaMag magnet	Invitrogen	12321D
Dynabeads™ FlowComp™ Mouse CD4 Kit	Invitrogen	11461D
Dynabeads™ FlowComp™ Mouse CD8 Kit	Invitrogen	11462D
Dynabeads™ Mouse T-Activator CD3/CD28 for T-Cell Expansion and Activation	ThermoFisher	11452D

Solutions, medium, supplement and enzymes

Product name	Company	Product number
Phosphate buffered Saline (PBS)	Gibco	10010-015
L-glutamine 200mM (100x)	Gibco	25030-024
RPMI Medium	Gibco	11835-063
Dulbecco's MEM	Merck	1469C
Fetales bovines Serum	Sigma-aldrich	S0615
MEM NEAA (100x)	Gibco	11140-035
Sodium-Pyruvate	Sigma	P2256
Penicillin-Streptomycin	Thermo Fisher	15140-122
Liberase	Roche	5401119001
Dnase I	Roche	10269638001
UltraPure™ 0.5M EDTA, pH 8.0	ThermoFisher	15575020
MACS Buffer BSA Stock Solution	Miltenyi Biotec	130-091-376
20% Glucosum	Bichsel	FE1001327
Normocin	InvivoGen	ant-nr-1
Cas9	Synthego	
electroporation enhancer	IDT	222501138
ROCK inhibitor Y27632	Lucerna Chem	MCE-HY-10583

Peptides, and Recombinant proteins

Product name	Company	Product number
Ovalbumin (323-339) (chicken, Japanese quail)	Sigma Aldrich	O1641-5MG
Recombinant Murine M-CSF	Peprotech	315-02
Recombinant Murine GM-CSF	Peprotech	315-03
20% Human Serum Albumin	CSL Berhing AG	3665734
Hemin	Sigma	51280
Bovine serum albumin	Newark	16009-13-5
Poly(I:C)	InvivoGen	tlrl-pic

Chemicals

Product name	Company	Product number
Formalin 10%, gepuffert	Formafix	01-1061
β-mercaptoethanol	Sigma-Aldrich	M3148-100ML
RBC Lysis Buffer (10X)	Biolegend	420301
Hematoxylin solution acc. to Gill II	Carl Roth	T864.2
Eosin Y w/ Phloxine solution	Epredia	71304
Mayer's Hematoxylin Solution	Sigma-Aldrich	51275-500ML
Geltrex™ LDEV-Free Reduced Growth Factor Basement Membrane Matrix	Thermo Fisher	A1413202
Phorbol-12-myristat-13-acetat	Sigma-Aldrich	P8139

Critical Commercial Assays

Product name	Company	Product number
UltraComp eBeads™ Compensation Beads	Thermo Fisher	01-2222-42
Tru Stain FcxTmPLUS CD16/32, clone S17011E Isotype Rat IgG2b	Biolegend	156604
Chromium Next GEM Single Cell Fixed RNA Sample Preparation Kit, 16 rxns	10x Genomics	1000414
Chromium Next GEM Chip Q Single Cell Kit, 16 rxns	10x Genomics	1000422
Dual Index Kit TS Set A, 96 rxn	10x Genomics	1000251
RNA screentape	Agilent Technologies	5067-5576
RNA ScreenTape Sample Buffer	Agilent Technologies	5067-5577
RNA ScreenTape Ladder	Agilent Technologies	5067-5578
Genomic DNA screentape	Agilent Technologies	5067-5365
Genomic DNA Reagents	Agilent Technologies	5067-5366
Chromium Fixed RNA Kit, Human Transcriptome, 4rxns x 4 BC	10x Genomics	1000475
Chromium Fixed RNA Kit, Mouse Transcriptome, 4rxns x 4 BC	10x Genomics	1000496
Visium CytAssist Spatial Gene Expression for FFPE, Mouse Transcriptome, 6.5mm, 4 rxns	10x Genomics	1000521
Visium CytAssist Spatial Gene Expression for FFPE, Human Transcriptome, 6.5mm, 4 rxn	10x Genomics	1000520
Visium HD, Mouse Transcriptome, 6.5 mm, 4 rxns	10x Genomics	1000676
Visium HD, Human Transcriptome, 6.5 mm, 4 rxns	10x Genomics	1000675
SYBR™ Green Master Mix	Applied Biosystems	4385612
Illumina® Stranded mRNA Prep, Ligation (96 Samples)	Illumina	20040534
RNeasy Mini Kit	Qiagen	74106
TaqMan reverse transcription reagents	Life Technologies	N8080234
VECTASTAIN® Elite ABC-HRP Kit, Peroxidase (Rabbit IgG)	Vector Laboratories	PK-6101
DAB Substrate Kit	abcam	ab64238
SG Cell Line 4D Nucleofector Kit	Lonza	V4XC-3032

Plates and coating

Product name	Company	Product number
96-well plates	Techno Plastic Products (TPP)	92096
12-well plates	Techno Plastic Products (TPP)	92012
6-well plates	Techno Plastic Products (TPP)	92006
6 cm culture dish	Techno Plastic Products (TPP)	93100
15 cm culture dish	Techno Plastic Products (TPP)	93150
Nunc™ Multidishes with UpCell™ Surface 6well	Thermo Fisher	174901
Nunc™ Dishes with UpCell™ Surface; 60mm	Thermo Fisher	174903
PrimeSurface® 3D culture Ultra-low Attachment Plates 96 well, U bottom, Clear plates	S-Bio	MS-9096UZ
SphericalPlate® 5D microwell, 24 well	Axon-Lab	12038828
96-well Clear Round Bottom Not Treated Microplate	Falcon®	351177

Primer sequences

Mouse	for	rev
Arg1	GTAGACCCTGGGGAACACTAT	ATCACCTTGCCAATCCCCAG
Cxcl10	GCTGCCGTCATTTTCTGC	TCTCACTGGCCCGTCATC
Gclm	AGTTGACATGGCATGCTCCG	CCATCTTCAATCGGAGGCGA
**Human**		
CXCL9	TGAGAAAGGGTCGCTGTTCC	GGGCTTGGGGCAAATTGTTT
GCLM	GGGAACCTGCTGAACTGGG	CTGGAAACTCCTCCTCGCTG.
HLA_DRB1	ACAGTTCCTCGGAGTGGAGA	AGCTCAGGAATCCTCTTGGC
SPP1	ACAAATACCCAGATGCTGTGGC	ACTTGGAAGGGTCTGTGGGG

sgRNAs

Synthego	sgRNA1	sgRNA2	sgRNA3
KEAP1	5’-ACCAACGGGCUGCGGGAGCA-3’	5’-UGGGCCAUGAACUGGGCGG[illegible]	5’-CCGUGUAGGCGAAUUCAAUG-3’
