# Supplementary figures and images for: Stress-NRF2 response axis polarizes tumor macrophages and undermines immunotherapy
Source: J Immunother Cancer. 2025 Oct 31;13(10):e013063. doi: 10.1136/jitc-2025-013063 (PMC12581087; doi:10.1136/jitc-2025-013063)

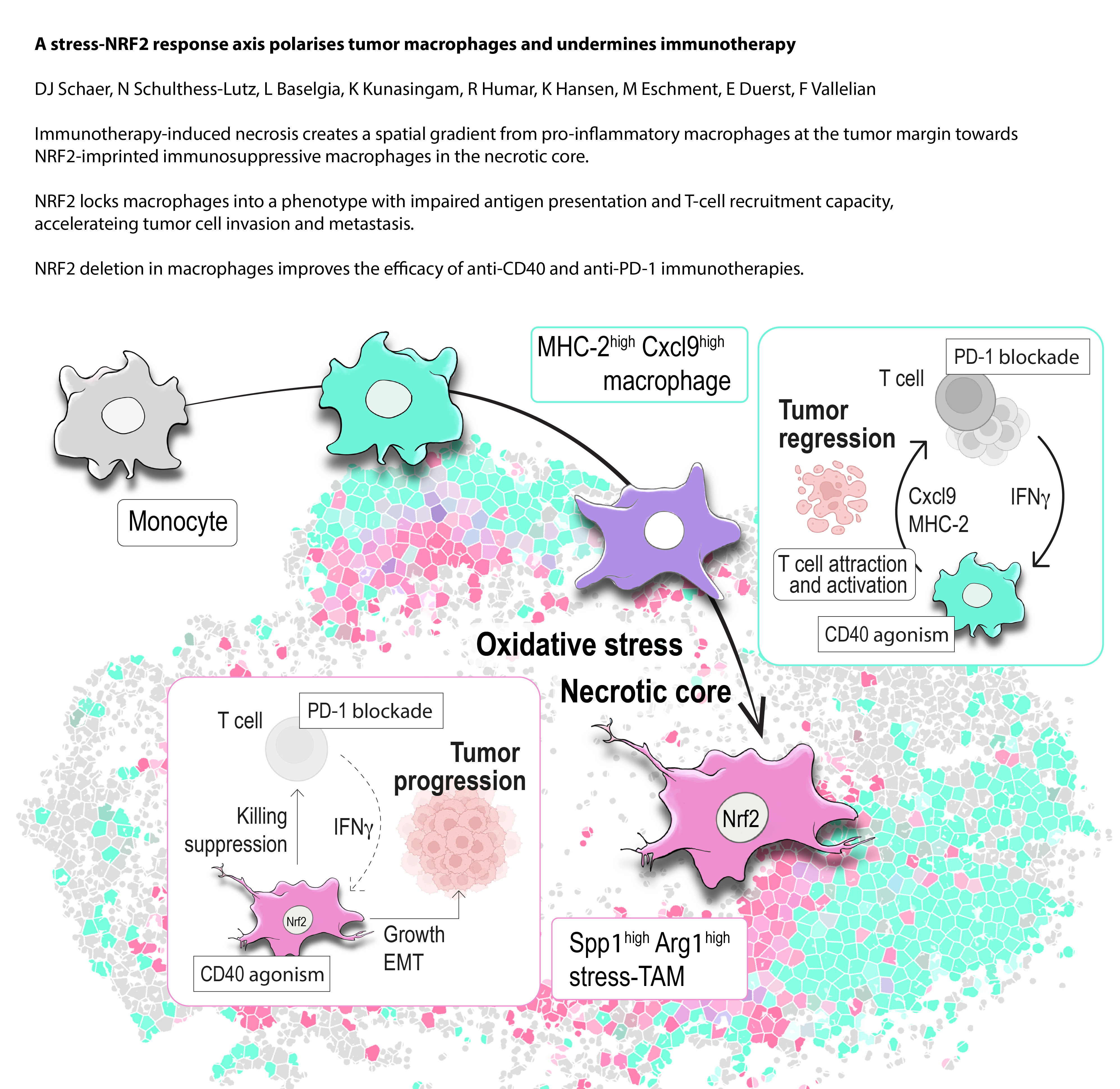

Supplement: online supplemental file 4 [file jitc-13-10-s004.tif]
